# The inference of sex-biased human demography from whole-genome data

**DOI:** 10.1371/journal.pgen.1008293

**Published:** 2019-09-20

**Authors:** Shaila Musharoff, Suyash Shringarpure, Carlos D. Bustamante, Sohini Ramachandran

**Affiliations:** 1 Department of Genetics, Stanford University, Stanford, CA, USA; 2 Center for Computational Molecular Biology, Brown University, Providence, RI, USA; 3 Ecology and Evolutionary Biology, Brown University, Providence, RI, USA; University of Pennsylvania, UNITED STATES

## Abstract

Sex-biased demographic events (“sex-bias”) involve unequal numbers of females and males. These events are typically inferred from the relative amount of X-chromosomal to autosomal genetic variation and have led to conflicting conclusions about human demographic history. Though population size changes alter the relative amount of X-chromosomal to autosomal genetic diversity even in the absence of sex-bias, this has generally not been accounted for in sex-bias estimators to date. Here, we present a novel method to identify sex-bias from genetic sequence data that models population size changes and estimates the female fraction of the effective population size during each time epoch. Compared to recent sex-bias inference methods, our approach can detect sex-bias that changes on a single population branch without requiring data from an outgroup or knowledge of divergence events. When applied to simulated data, conventional sex-bias estimators are biased by population size changes, especially recent growth or bottlenecks, while our estimator is unbiased. We next apply our method to high-coverage exome data from the 1000 Genomes Project and estimate a male bias in Yorubans (47% female) and Europeans (44%), possibly due to stronger background selection on the X chromosome than on the autosomes. Finally, we apply our method to the 1000 Genomes Project Phase 3 high-coverage Complete Genomics whole-genome data and estimate a female bias in Yorubans (63% female), Europeans (84%), Punjabis (82%), as well as Peruvians (56%), and a male bias in the Southern Han Chinese (45%). Our method additionally identifies a male-biased migration out of Africa based on data from Europeans (20% female). Our results demonstrate that modeling population size change is necessary to estimate sex-bias parameters accurately. Our approach gives insight into signatures of sex-bias in sexual species, and the demographic models it produces can serve as more accurate null models for tests of selection.

## Author summary

Sex-biased demographic events involve unequal numbers of females and males, and is referred to as “sex-bias”. In humans, short-range migrations (e.g., due to marriage practices) are known to be sex-biased, and some long-range migrations, such as the one out of Africa, are hypothesized to be sex-biased. The recent availability of large-scale genomic sequencing data provides a unique opportunity to study sex-bias in human populations. However, existing sex-bias methods do not account for population size changes, like expansions and bottlenecks, or can only estimate a single sex-bias parameter on a population branch, which can lead to incorrect conclusions. We developed a sex-bias method which explicitly models population size changes, and we show that it outperforms competing methods on simulated data. When applied to human genetic data, our method identifies an overall female sex-bias in globally-distributed populations and a male-biased bottleneck in Europeans. Our method can also be used to assess sex-bias in other sexual species.

## Introduction

Human population-genetic studies generally assume that the proportions of reproducing females and males are equal. However, human history contains sex-biased demographic events (“sex-bias”) which are defined by having unequal female and male effective population sizes, $N_{e}^{F}$ and $N_{e}^{M}$. Some examples of sex-bias include matrilocality (the practice of females remaining in their place of birth after marriage), and conversely, patrilocality [1, 2]; patrilineal inheritance in herder groups [3]; polygamy, the practice of a male having multiple female sexual partners, and polyandry, which is the opposite; female- and male-biased migration; and sexual selection. These factors, along with a variance in reproductive success that is greater in males than females [4, 5], cause male and female effective sizes to differ [6, 7].

Initial studies of human sex-bias compared mitochondrial to Y-chromosomal data due to their uniparental inheritance (maternal and paternal, respectively). Recent studies have compared X-chromosomal to autosomal data [8–11] to take advantage of their multiple independent loci [12, 13]. Most of these studies found evidence for female bias in human populations. Although Labuda et al. initially found evidence for male bias based on recombination rates [14], their conclusion changed to one of a female bias after an error in their analysis was corrected [15, 16]. These studies used standard sex-bias estimators of *Q*, the ratio of X-chromosomal to autosomal effective population sizes. In a neutrally-evolving population of constant size with no migration, *Q* is 0.75 when there is no sex-bias; *Q* is greater than 0.75 when there is a female bias and less than 0.75 when there is a male bias. Other recent sex-bias studies analyzed admixture fraction on the X-chromosome and autosomes and found evidence for sex-biased admixture in human populations.

Since they have different effective sizes, the X chromosome and autosomes recover genetic diversity following a population size change at different rates, even in the absence of sex-bias [17]. Previous studies considered whether population size change alone could explain the patterns of X-chromosomal and autosomal genetic variation observed in human populations. Though a study of genomic resequencing data estimated a large *Q* value consistent with a bottleneck more than 100,000 years ago followed by recent growth, they rejected this explanation based on simulations [18]. A more recent study, which found that *Q* increases with distance from genes, studied the impact of human demographic histories on ${\hat{Q}}_{\pi }$ (i.e., *Q* estimated from *π*, average pairwise sequence diversity) and found it was only slightly biased by realistic size changes [19]. The common estimators of sex-bias, ${\hat{Q}}_{\pi }$ and ${\hat{Q}}_{F_{ST}}$, *Q* estimated from *F*_*ST*_, are sensitive to sex-biases at different time scales in the context of realistic human demographic history [20]. A study assessing a male-biased Out-of-Africa bottleneck found evidence for a more severe bottleneck on the X chromosome than the autosomes in European and East Asians but was not able to estimate the proportion of females during the bottleneck with their *F*_*ST*_-based inference method [21, 22]. Although these studies characterized patterns of relative X-chromosomal to autosomal variation, they did not explicitly model population size change, nor did they provide estimates of the proportion of females during specific epochs. A recent study by Clemente et al. developed a tree-based method, KimTree, to estimate sex-bias parameters for each population branch from multi-population X-chromosomal and autosomal data [23]. Although they found evidence for an overall female bias in human populations and a male bias in Oceanians, their inference could be biased for one of the following reasons, among others: their method assumes a constant population size during epochs, and they did not remove genic regions from their human data, which could be under evolutionary constraints. Furthermore, their method requires data from multiple populations, limiting its applicability.

Here, we present a novel method to estimate sex-bias from X-chromosomal and autosomal sequence data. It models demographic history jointly from X-chromosomal and autosomal site frequency spectra and explicitly models complex demographic events, including exponential growth and multiple bottlenecks. Our method estimates the proportion of females overall as well as during each time epoch. In simulations, our method has good power to detect a true sex-bias for a range of demographic histories and performs well when the method of Clemente et al. does not. We apply our method to globally-distributed human populations from the 1000 Genomes Project [24] and compare sex-bias estimates based on exome data to those from whole-genome sequencing data. Our sex-bias estimates, which account for population size changes, give new insight into human demographic history and the male-biased migration out of Africa.

## Results

First, we present a framework to infer sex-biased demography while modeling population size changes. Next, we apply our method to data simulated under one of three demographic models: constant size, a two-epoch expansion, or a three-epoch bottleneck. Finally, we apply our method to exome and whole-genome sequence data from the 1000 Genomes Project.

### Theoretical framework: Population of constant size

We initially assume a constant per-site mutation rate, *μ*, shared by the X chromosome and autosomes, and that mutation occurs as a Poisson process [25]. We later account for unequal male and female mutation rates. For a population with *N*_*m*_ males and *N*_*f*_ females where *N*_*m*_ + *N*_*f*_ = *N*, the total number of individuals, the inbreeding effective sizes of the autosomes and X chromosome can be derived using a coalescent argument [13]. In terms of the proportion of breeding females *p* = *N*_*f*_/*N*, these effective sizes are:

$$\begin{array}{c} N_{e}^{A}\left(p\right)=4p\left(1-p\right)N \end{array}$$ (1)

$$\begin{array}{c} N_{e}^{X}\left(p\right)=\frac{9p(1-p)}{2(2-p)}N \end{array}$$ (2)

**Fig 1:**
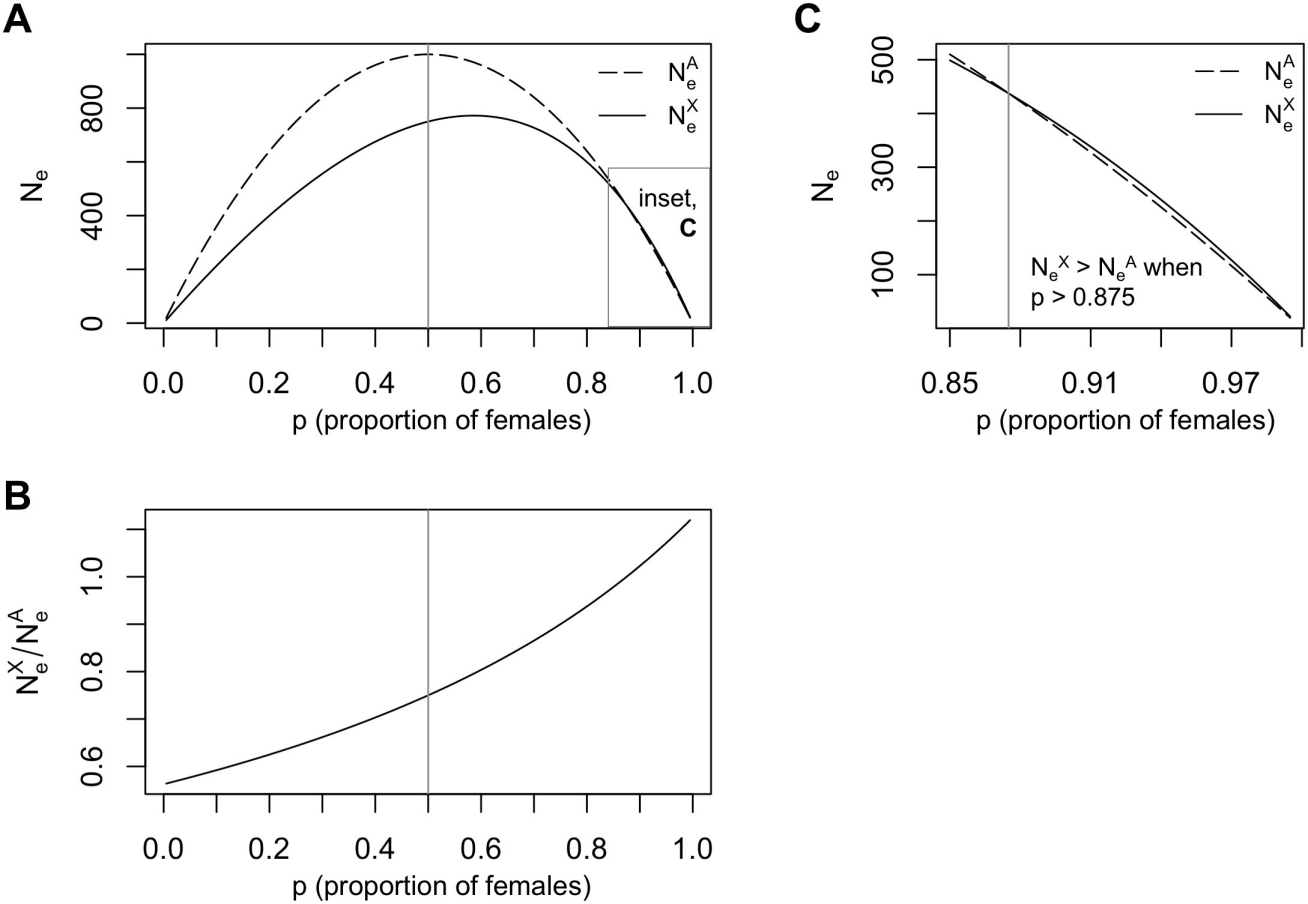
Effective population size and sex-bias. (A) Expected autosomal ($N_{e}^{A}$) and X-chromosomal ($N_{e}^{X}$) effective population sizes as a function of the proportion females (*p*) for *N* = 1000 individuals. When *p* = 0.5, there is no sex-bias, as denoted by the gray line. (B) The ratio of X-chromosomal to autosomal effective sizes, $N_{e}^{X}/N_{e}^{A}$, increases with *p*. This ratio is 0.75 when there is no sex-bias (*p* = 0.5, gray line) and is undefined when *p* is 0 or 1. (C) Inset of A: when *p* is greater than 0.875 (to the right of the gray line), $N_{e}^{X}$ is greater than $N_{e}^{A}$, so $N_{e}^{X}/N_{e}^{A}$ is greater than 1.

We drop *p* from the left-hand side for notational convenience. The autosomal effective size ($N_{e}^{A}$) is maximized at *N* when *p* = 0.5 and is less than *N* otherwise; the X-chromosomal effective size ($N_{e}^{X}$) is always less than *N* (Fig 1A). We define these reductions of effective size due to sex-bias (i.e. *p* ≠ 0.5) as the reduction factors $f_{A}\left(p\right)=N_{e}^{A}/N$ and $f_{X}\left(p\right)=N_{e}^{X}/N$. The ratio $N_{e}^{X}/N_{e}^{A}$ increases with *p* (Fig 1B) and is greater than 1 for very female biased values (*p* > 0.875), in agreement with classic results (Fig 1C).

The unfolded site frequency spectrum (SFS) for a sample of *n* chromosomes is the random vector (*S*_1_, *S*_2_, …, *S*_*n*−1_). Under the Poisson random field model, the *S*_*i*_’s are independent Poisson-distributed entries with mean *θF*(*i*) (see Eq 2 of [25] for the definition of *F*(*i*)). The probability of observing *s*_*i*_ sites with *i* derived and (*n* − *i*) ancestral mutations under neutrality, given a population-scaled mutation rate of *θ* = 4*N*_*e*_*μ* and the demographic model *D*, is:

$$\begin{array}{c} p\left(S_{i}=s_{i}|\theta ,D\right)=e^{-\theta F(i)}\frac{{\left(\theta F\left(i\right)\right)}^{s_{i}}}{s_{i}!} \end{array}$$ (3)

The maximum likelihood estimator (MLE) of *θ* for a sample of *n* chromosomes with a total of *S* segregating sites (i.e. $S=\sum_{i=1}^{n}S_{i}$) is as follows, where the subscripts *A* and *X* denote the autosomes and X chromosome, respectively (see also Eq 13 of [25]):

$$\begin{array}{c} {\hat{\theta }}_{A}=\frac{S_{A}}{\sum_{i=1}^{n_{A}-1}F_{A}\left(i\right)} \end{array}$$ (4)

$$\begin{array}{c} {\hat{\theta }}_{X}=\frac{S_{X}}{\sum_{j=1}^{n_{X}-1}F_{X}\left(j\right)} \end{array}$$ (5)

To test for sex-bias in a population of constant size, we develop a likelihood ratio test (LRT). Under the null hypothesis, the parameters ${\hat{\theta }}_{A}$ and ${\hat{\theta }}_{X}$ are consistent with *p* = 0.5; under the alternate hypothesis, they are not. Let *L*_*A*_ and *L*_*X*_ be the physical length (e.g., in base pairs) of the sequenced autosomal and X-chromosomal loci. The Poisson density in Eq 3 can be combined with the MLEs in Eqs 4 and 5 to give distributions of the number of autosomal and X-chromosomal segregating sites:

$$\begin{array}{c} S_{A}∼Pois\left(\theta_{A}\times L_{A}\times \sum_{i=1}^{n_{A}-1}F_{A}\left(i\right)\right) \end{array}$$ (6)

$$\begin{array}{c} S_{X}∼Pois\left(\theta_{X}\times L_{X}\times \sum_{j=1}^{n_{X}-1}F_{X}\left(j\right)\right) \end{array}$$ (7)

We combine the definition *θ* = 4*N*_*e*_*μ* with Eqs 1 and 6 to get the expectation of *S*_*A*_ and with Eqs 2 and 7 to get the expectation of *S*_*X*_:

$$\begin{array}{c} E\left[S_{A}\right]=4p\left(1-p\right)N\mu \times L_{A}\times \sum_{i=1}^{n_{A}-1}F_{A}\left(i\right) \end{array}$$ (8)

$$\begin{array}{c} E\left[S_{X}\right]=\frac{9p(1-p)}{2(2-p)}N\mu \times L_{X}\times \sum_{j=1}^{n_{X}-1}F_{X}\left(j\right) \end{array}$$ (9)

Taking the ratio of *S*_*A*_ to *S*_*X*_ and solving for *p*, we obtain our estimator of the effective proportion of females overall, $\tilde{p}$, in terms of the site frequency spectrum densities *F*_*A*_ and *F*_*X*_:

$$\begin{array}{c} \tilde{p}=2-\frac{9S_{A}L_{X}\sum_{j=1}^{n_{X}-1}F_{X}\left(j\right)}{8S_{X}L_{A}\sum_{i=1}^{n_{A}-1}F_{A}\left(i\right)} \end{array}$$ (10)

To estimate $\tilde{p}$ for a particular epoch *t*, we use the effective sizes $N_{et}^{A}$ and $N_{et}^{X}$ for that epoch:

$$\begin{array}{c} \tilde{p_{t}}=2-\frac{9\times 4N_{et}^{A}\mu L_{A}}{8\times 4N_{et}^{X}\mu L_{X}} \end{array}$$ (11)

Using Eqs 6 and 7, we can write a joint likelihood for the autosomal and X-chromosomal data:

$$\begin{array}{cc} L(\theta ,p|S_{A},S_{X}) & =\prod_{i=1}^{n_{A}-1}P\left(S_{i,A}=s_{i,A}|\theta_{A}\right)\prod_{i=1}^{n_{X}-1}P\left(S_{i,X}=s_{i,X}|\theta_{X}\right)\\ & =\prod_{i=1}^{n_{A}-1}e^{-\theta_{A}F_{A}\left(i\right)}\frac{{\left(\theta_{A}F_{A}\left(i\right)\right)}^{s_{i,A}}}{s_{i,A}!}\prod_{i=1}^{n_{X}-1}e^{-\theta_{X}F_{X}\left(i\right)}\frac{{\left(\theta_{X}F_{X}\left(i\right)\right)}^{s_{i,X}}}{s_{i,X}!} \end{array}$$ (12)

For a population of constant size, this likelihood reduces to the likelihood in [18], which we can use to define a likelihood ratio test for sex-bias. Under the null hypothesis of *p* = 0.5 (i.e. no sex-bias), *θ*_*A*_ = *θ* and *θ*_*X*_ = 3/4 × *θ* based on Eqs 1 and 2. Substituting these into Eq 12, we obtain the MLE of *θ* based on autosomal and X-chromosomal data under the null hypothesis:

$$\begin{array}{c} {\tilde{\theta }}_{0}=\frac{S_{A}+S_{X}}{L_{A}\times \sum_{i=1}^{n_{A}-1}F_{A}\left(i\right)+3/4\times L_{X}\times \sum_{j=1}^{n_{X}-1}F_{X}\left(j\right)} \end{array}$$ (13)

Under the alternative hypothesis of sex-bias (*p* ≠ 0.5), we instead use the reduction factors in *θ*_*A*_ = *f*_*A*_(*p*) × *θ* and *θ*_*X*_ = *f*_*X*_(*p*) × *θ* and obtain the MLE of *θ* as:

$$\begin{array}{c} \tilde{\theta }=\frac{S_{A}+S_{X}}{f_{A}\left(p\right)\times L_{A}\times \sum_{i=1}^{n_{A}-1}F_{A}\left(i\right)+f_{X}\left(p\right)\times L_{X}\times \sum_{j=1}^{n_{X}-1}F_{X}\left(j\right)} \end{array}$$ (14)

We evaluate the log of the likelihood in Eq 12 at $\theta ={\tilde{\theta }}_{0}$ to obtain the null log-likelihood, *LL*_0_, and at $\theta =\tilde{\theta }$ to obtain *LL*_1_, the alternative log-likelihood. The likelihood ratio test statistic, Λ = −2(*LL*_0_ − *LL*_1_), is approximately $\chi_{1}^{2}$-distributed.

### Theoretical framework: Population of non-constant size

We define a demographic history as a set of population sizes (*N*_*e*1_, *N*_*e*2_, …*N*_*eT*_) which go forward in time (i.e., *N*_*e*1_ is the ancestral population size) and correspond to a set of *T* − 1 size changes and *T* epoch durations. The size changes $\vec{\nu }=\left(\nu_{1},\nu_{2},\ldots ,\nu_{T-1}\right)$, which occur instantaneously or exponentially, are defined as the size at the end of an epoch relative to the ancestral population size. The epoch durations $\vec{\tau }=\left(\tau_{1},\tau_{2},\ldots ,\tau_{T}\right)$ are in units of genetic time scaled by the ancestral population size. We assume the X chromosome has the same demographic model (i.e. number and kind of size changes) as the autosomes. To assess sex-bias in a population, we test nested X-chromosomal models defined in terms of the female fraction of the effective size during an epoch, *p*_*t*_, *t* = 1…*T*:

**Model 0**: no sex-bias. *p* is 0.5 for every epoch, so $N_{t}^{X}=0.75\times N_{t}^{A}$.
**Model 1**: constant sex-bias. *p*_*t*_ is the same for every epoch, so $N_{t}^{X}=c\times N_{t}^{A}$ for a constant *c*.
**Model *T***: varying levels of sex-bias. *p*_*t*_ can vary among epochs, so $N_{t}^{X}=c_{t}\times N_{t}^{A}$ for a constant *c*_*t*_.

These models are implemented by constraining the X-chromosomal size change and epoch duration parameters, $\vec{\nu^{X}}$ and $\vec{\tau^{X}}$, by the autosomal parameters $\vec{\nu^{A}}$ and $\vec{\tau^{A}}$, and their likelihoods are used in the likelihood ratio tests for sex-bias (see S1 Text, “Likelihood ratio tests for sex-bias: general form”). In addition to the examples we give for a two-epoch model below (“Sex-bias tests for a two-epoch model”) and a three-epoch bottleneck model (see S1 Text, “Likelihood ratio tests for sex-bias: bottleneck model”), sex-bias tests for arbitrarily complex demographic models can be defined.

### Sex-bias tests for a two-epoch model

**Fig 2:**
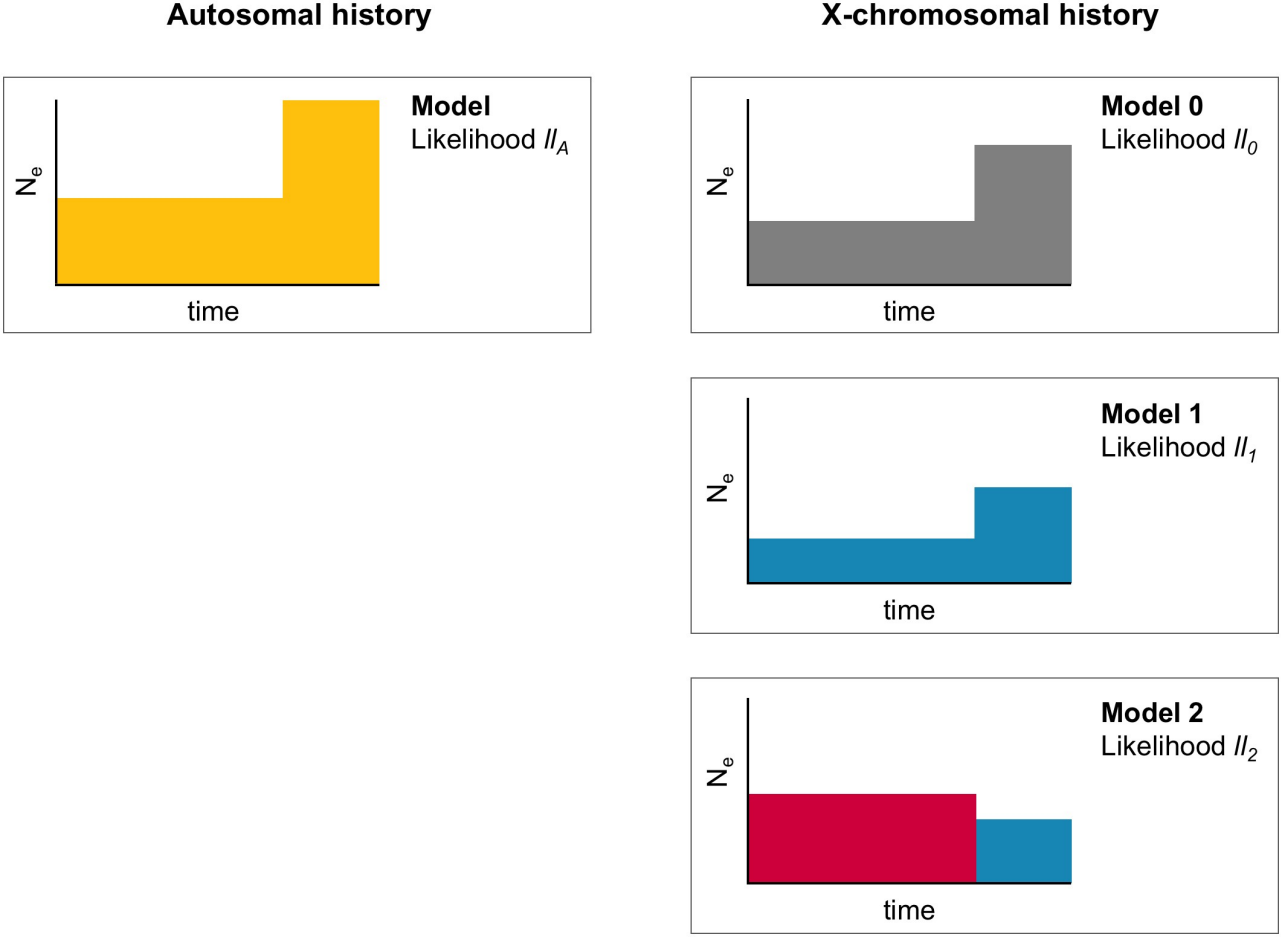
Sex-bias tests for a two-epoch demographic model. Each plot is a demographic model with time on the x-axis and effective population size on the y-axis. For a two-epoch model, there are three possible X-chromosomal models, one for each sex-bias scenario: Model 0 has no sex-bias (*p* = 0.5), Model 1 has a constant sex-bias (*p* ≠ 0.5), and Model 2 has a changing sex-bias. The likelihoods of the autosomal and X-chromosomal models are used in the likelihood ratio tests. In these examples, epochs in gray have no sex-bias, those in blue have a male bias, and those in red have a female bias.

A population at mutation-drift equilibrium changes from its original size of *N*_0_ to size *N*_1_ (i.e. the fold-size change *ν* is *N*_1_/*N*_0_) at time *τ* (Fig 2). Though a population expansion is shown in the figure, the same framework is used for a population contraction. There are two free X-chromosomal parameters, *ν*_*X*_ and *τ*_*X*_, so there are three X-chromosomal models, Models 0, 1, and 2, and two likelihood ratio tests. Model 0 has no sex-bias (*p* = 0.5) and the following constraints ensure that the effective size of the X chromosome is 3/4 that of the autosomes: *ν*_*X*_ = *ν*_*A*_, *τ*_*X*_ = 4/3 × *τ*_*A*_, *θ*_*X*_ = 4/3 × *θ*_*A*_. Model 1 has a constant sex-bias (*p* is constant) and these constraints ensure that the X-chromosomal effective sizes are a constant scaling of the autosomal effective sizes: *ν*_*X*_ = *ν*_*A*_, *τ*^*X*^ = 1/*c* × *τ*^*A*^, and *θ*^*X*^ = *c* × *θ*^*A*^ for some constant *c*. The final model, Model 2, corresponds to varying sex-bias (*p* varies), and its constraints ensure that the size changes happen at the same time as measured in generations: *ν*^*X*^ = *c*_2_/*c*_1_ × *ν*^*A*^, *τ*^*X*^ = 1/*c*_2_ × *τ*^*A*^, *θ*^*X*^ = *c*_1_ × *θ*^*A*^.

Joint likelihoods for the *i*^*th*^ model, *i* = 0, 1, 2, based on the autosomal log-likelihood *ll*_*A*_ and X-chromosomal log-likelihood *ll*_*i*_, are: *LL*_0_ = *ll*_*A*_ + *ll*_0_, *LL*_1_ = *ll*_*A*_ + *ll*_1_, and *LL*_2_ = *ll*_*A*_ + *ll*_2_. A test for constant sex-bias is based on Λ_0_ = −2 × (*LL*_0_ − *LL*_1_) and one for varying sex-bias is based on Λ_1_ = −2 × (*LL*_1_ − *LL*_2_). The best-fitting model has an estimate of the effective proportion of females overall, $\tilde{p}$, and during each epoch, $\tilde{p_{t}}$, *t* = 1…*T*.

### Results from simulations

#### Constant population size

**Fig 3:**
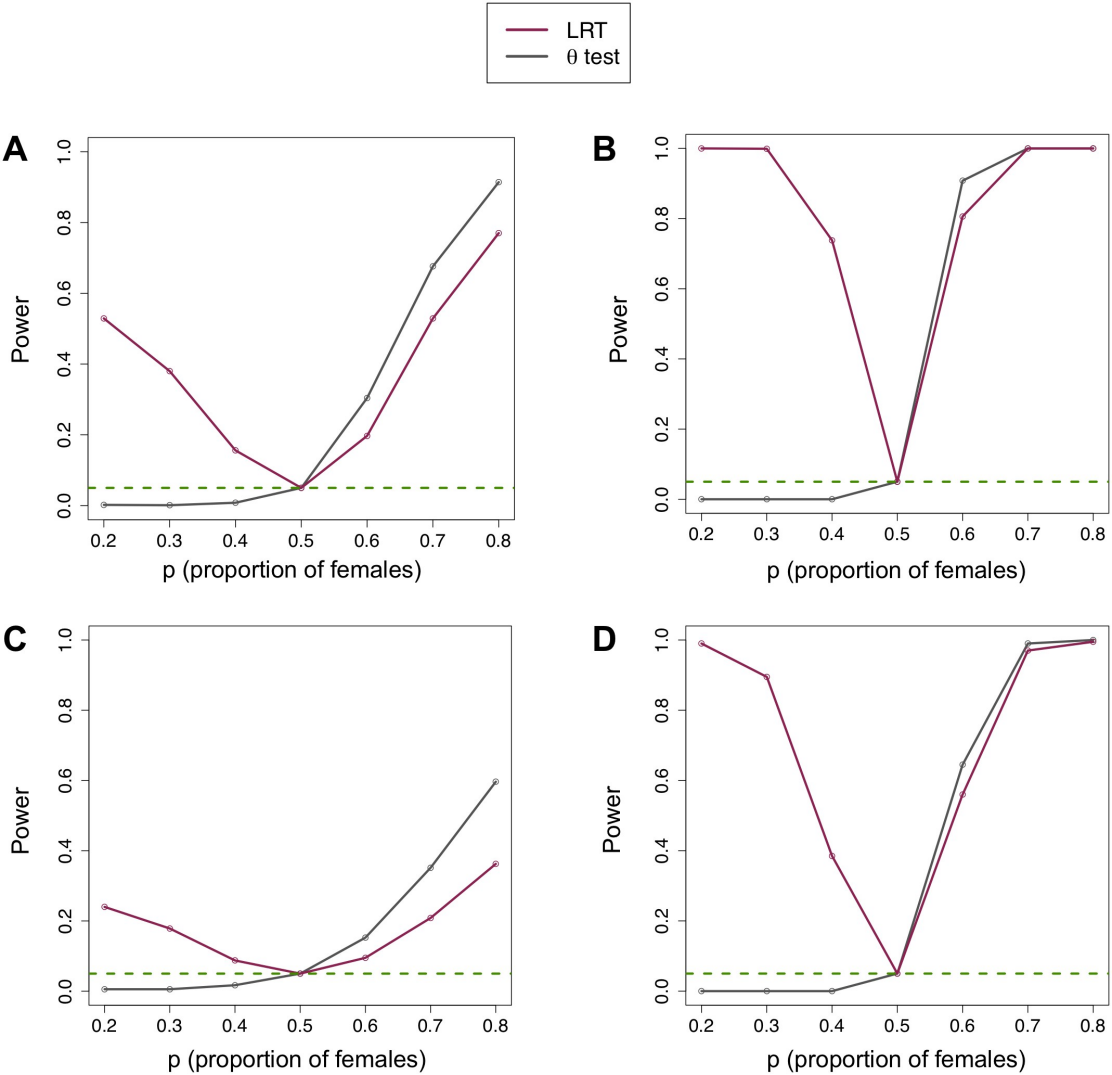
Sex-bias tests applied to a simulated population of constant size. Power of the likelihood ratio test for sex-bias (“LRT”) compared to a standard (“*θ* test”) applied to unlinked SNPs for (A) a small number of segregating sites (427) and (B) a large number of segregating sites (4253). Power of tests applied to partially linked SNPs for (C) a small number (637) and (D) a large number (6367) of segregating sites. The dashed green line is at the false positive rate of 0.05.

We simulated single nucleotide polymorphism (SNP) data at independent sites from a population of constant size (for details, see Materials and methods). On data simulated under the null (*p* = 0.5), our test for sex-bias is calibrated and our estimators of *p* and *θ* are unbiased (S1 Fig). On data simulated under the alternative (*p* = 0.2), our test has good power (S2 Fig). The power of our test for sex-bias (denoted “LRT”) and a test based on the standard estimator *Q* (denoted “*θ* test”) is in Fig 3. For a small number of segregating sites (an average of 427), our test has moderate power overall; although the *Q*-based test has better power to detect a female bias, it has almost no power to detect a male bias (Fig 3A). Increasing the number of segregating sites to an average of 4250 sites increases the power of both tests (Fig 3B). Both tests have less power on data simulated with linkage characteristic of human populations (Fig 3C and 3D), as expected.

#### Population expansion

**Fig 4:**
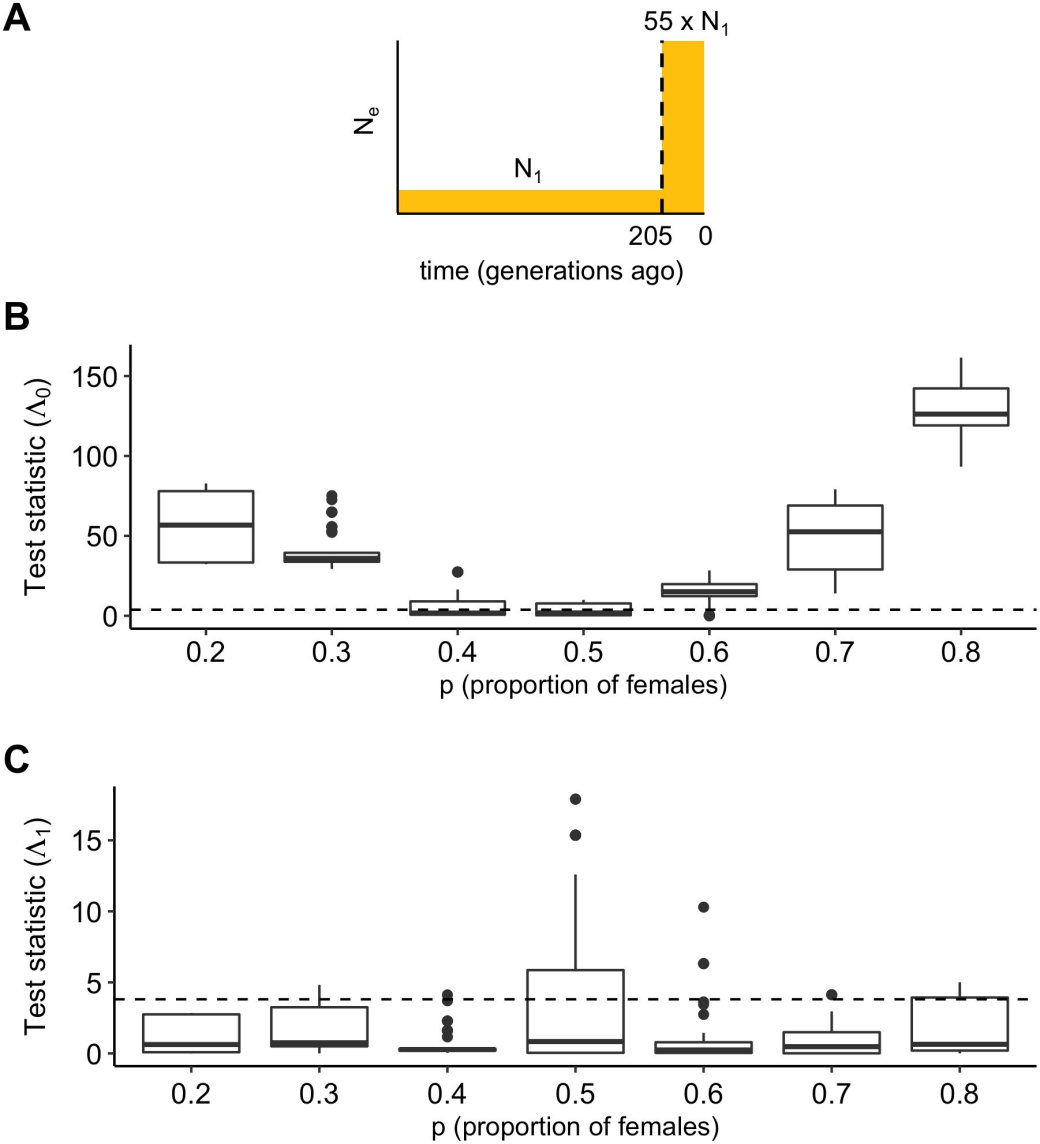
Test statistics for simulated recent growth. (A) A demographic model with recent growth (55x, 205 generations ago) was used to simulate data with a constant sex-bias for a sample of 500 chromosomes with independent sites. Test statistics for (B) a constant sex-bias, Λ_0_, and (C) changing sex-bias, Λ_1_, are shown for a range of the proportion of females, *p*. The critical value is denoted by the dashed line.

**Fig 5:**
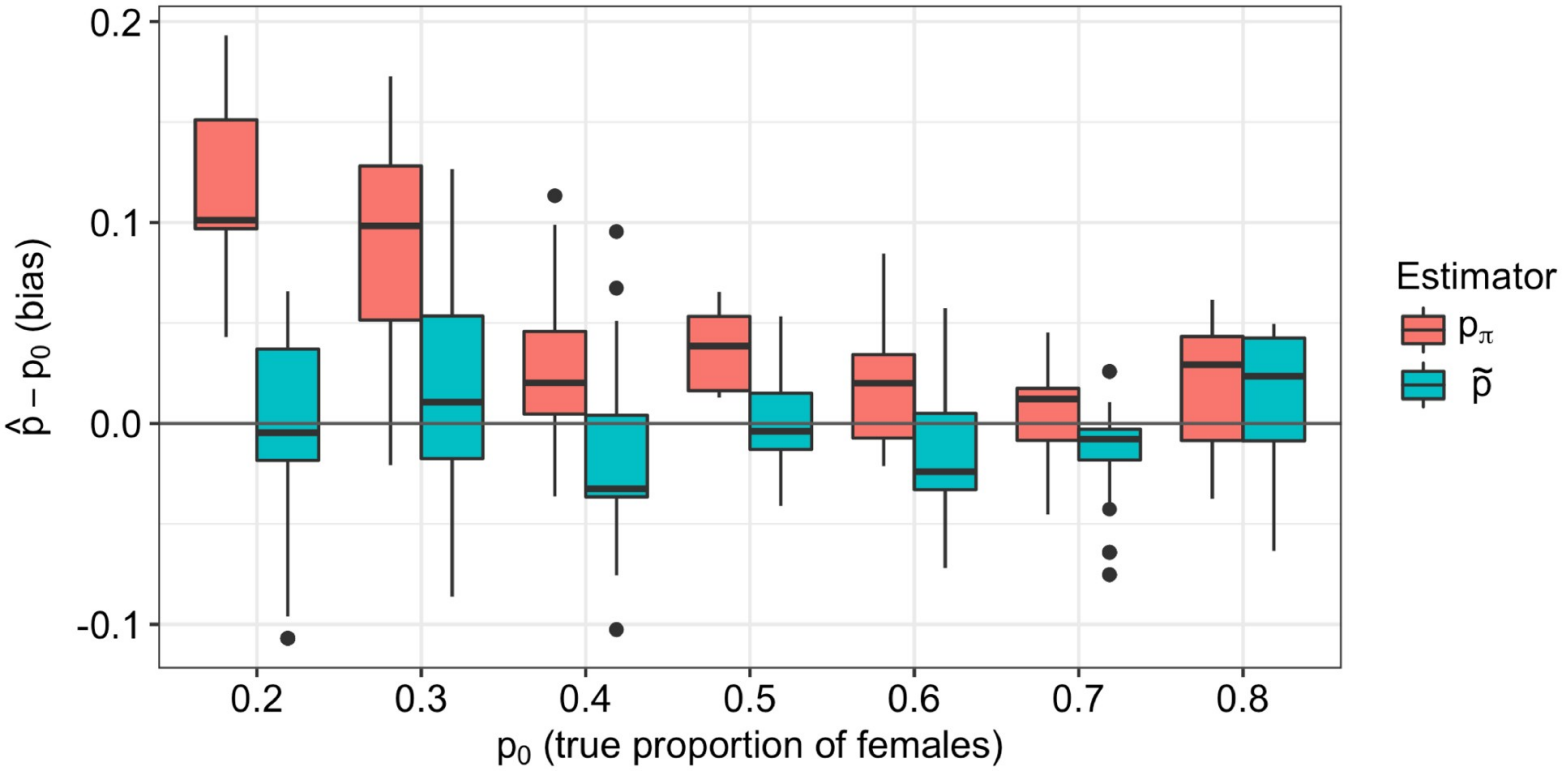
Estimates of *p* for simulated recent growth. Bias of sex-bias estimators applied to data simulated with recent growth (55x, 205 generations ago) and a constant sex-bias. The true proportion of females, *p*_0_, is on the x-axis and the bias of the estimator, $\hat{p}-p_{0}$, is on the y-axis. Our estimator, $\tilde{p}$, models population size change and is unbiased; the estimator *p*_*π*_, which is based on pairwise sequence diversity *π*, does not model population size change and is biased for small values of *p*_0_.

Population expansions are characteristic of recent human history and perturb the ratio of X-chromosomal to autosomal genetic diversity, even in the absence of sex-bias [17]. We simulated a sample of 500 chromosomes from a population which underwent a 55-fold expansion 205 generations ago (Fig 4A), which are parameters estimated from European genetic data [26]. We simulated either a constant sex-bias or no sex-bias for values of *p* from 0.2 to 0.8. Applied to this data, our test for constant sex-bias is well-powered (Fig 4B), except when *p* = 0.4. This is because the expected value of $N_{e}^{X}/N_{e}^{A}$ is 0.703 when *p* = 0.4, which is close its expected value of 0.75 when *p* = 0.5 under the null (Eqs 1 and 2). This test has less power on a smaller sample of 40 chromosomes (S3A Fig) and more power on a larger sample of 5000 chromosomes (S4A Fig). Our test for changing sex-bias is unbiased on 500 chromosomes (Fig 4C) as well as on 40 or 5000 chromosomes (S3B and S4B Figs). We next compared our estimator of the proportion of females, $\tilde{p}$, to the estimator *p*_*π*_, which is calculated from pairwise sequence diversity (*π*) of the autosomes and X chromosome [18] and is biased by population size change, even in the absence of sex-bias [17]. Applied to simulated data, our estimator $\tilde{p}$ is unbiased for all values of *p* because it accounts for population expansion (Fig 5). Conversely, *p*_*π*_ is biased under the null (*p* = 0.5) and performs poorly for small values of *p*: for a strong male bias of *p* = 0.2, the median *p*_*π*_ estimate is 0.305.

#### Population bottleneck

**Fig 6:**
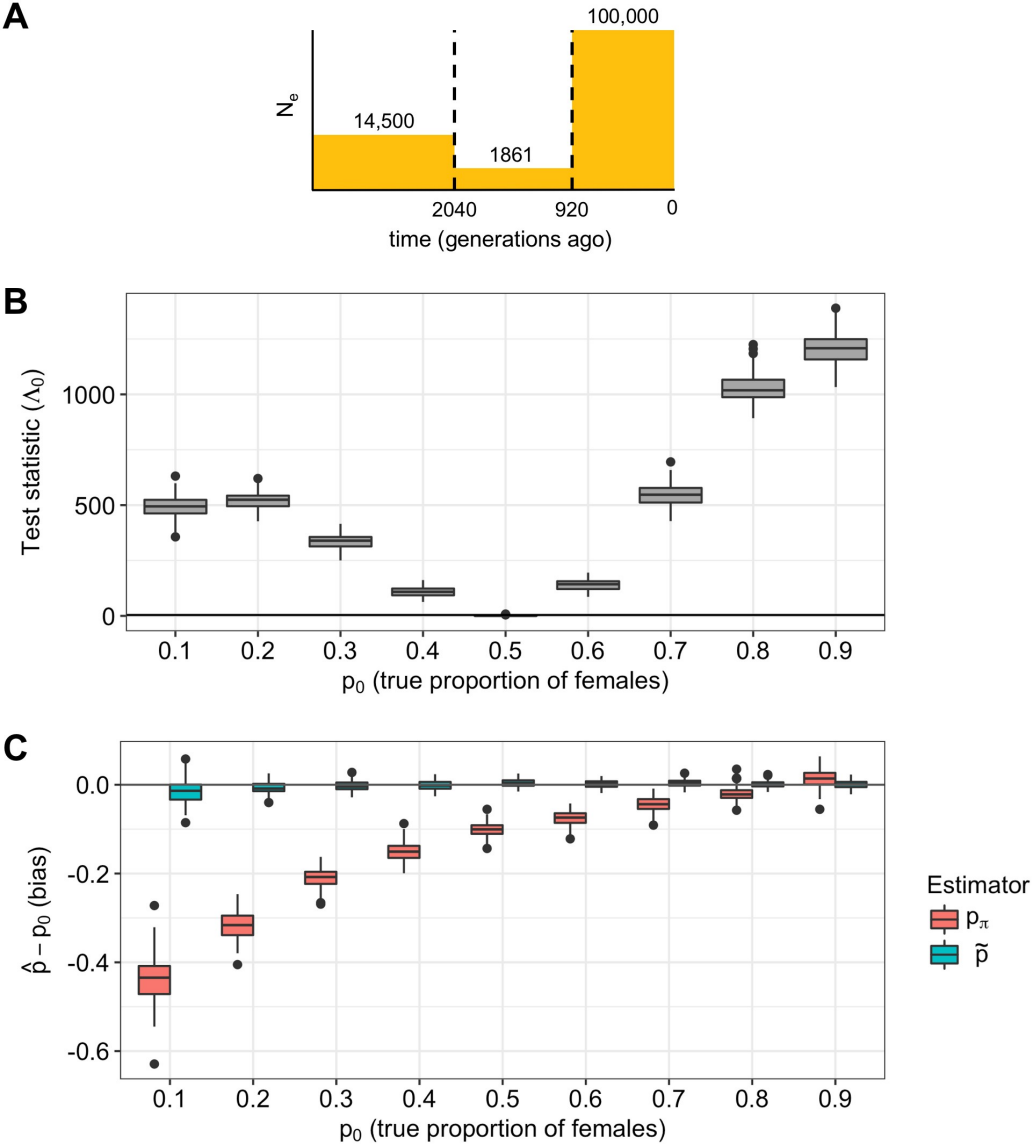
Simulated bottleneck with constant sex-bias. Bottleneck simulations with a constant proportion of females, *p*_0_. (A) Autosomal demographic model with time on the x-axis and effective population size on the y-axis. (B) Test statistics for the test for constant sex-bias, Λ_0_, with the parametric bootstrap critical value in gray. (C) Bias of estimators of the proportion of females, $\hat{p}-p_{0}$, is shown for our estimator, $\tilde{p}$, and the estimator based on pairwise sequence diversity, *p*_*π*_. Our estimator is unbiased while the *p*_*π*_ is increasingly biased for small *p*_0_ and at times negative.

The bottleneck model in Fig 6A is a simplified version of the Out-of-Africa bottleneck estimated from European individuals [26]. We first simulated a sample of 100 chromosomes under this model with a constant sex-bias. Applied to this data, our test for constant sex-bias has large LRT statistics Λ_0_ (Fig 6B) and a power of 1 for all values of *p* due to the large number of simulated segregating sites (approximately 20,000 sites for autosomal data). Our test for changing sex-bias has small test statistics Λ_1_ and a negligible false positive rate for all values of *p*. Our estimator of the overall effective proportion of females $\tilde{p}$ is unbiased (Fig 6C, blue). The estimator *p*_*π*_ works well when *p* = 0.8, but becomes increasingly biased as *p* decreases: for the strong male bias of *p* = 0.2, the median of *p*_*π*_ is -0.12 (Fig 6C, red). The *p*_*π*_-based test might have low power for small *p* because $N_{e}^{X}/N_{e}^{A}$ is perturbed less from its expected value by a reduction from 0.5 than the analogous increase: for example, $N_{e}^{X}/N_{e}^{A}$ is perturbed less from 0.75 when *p* = 0.2 than when *p* = 0.8 (Fig 1A). This results in an asymmetrical *p*_*π*_ bias curve (Fig 6C).

**Fig 7:**
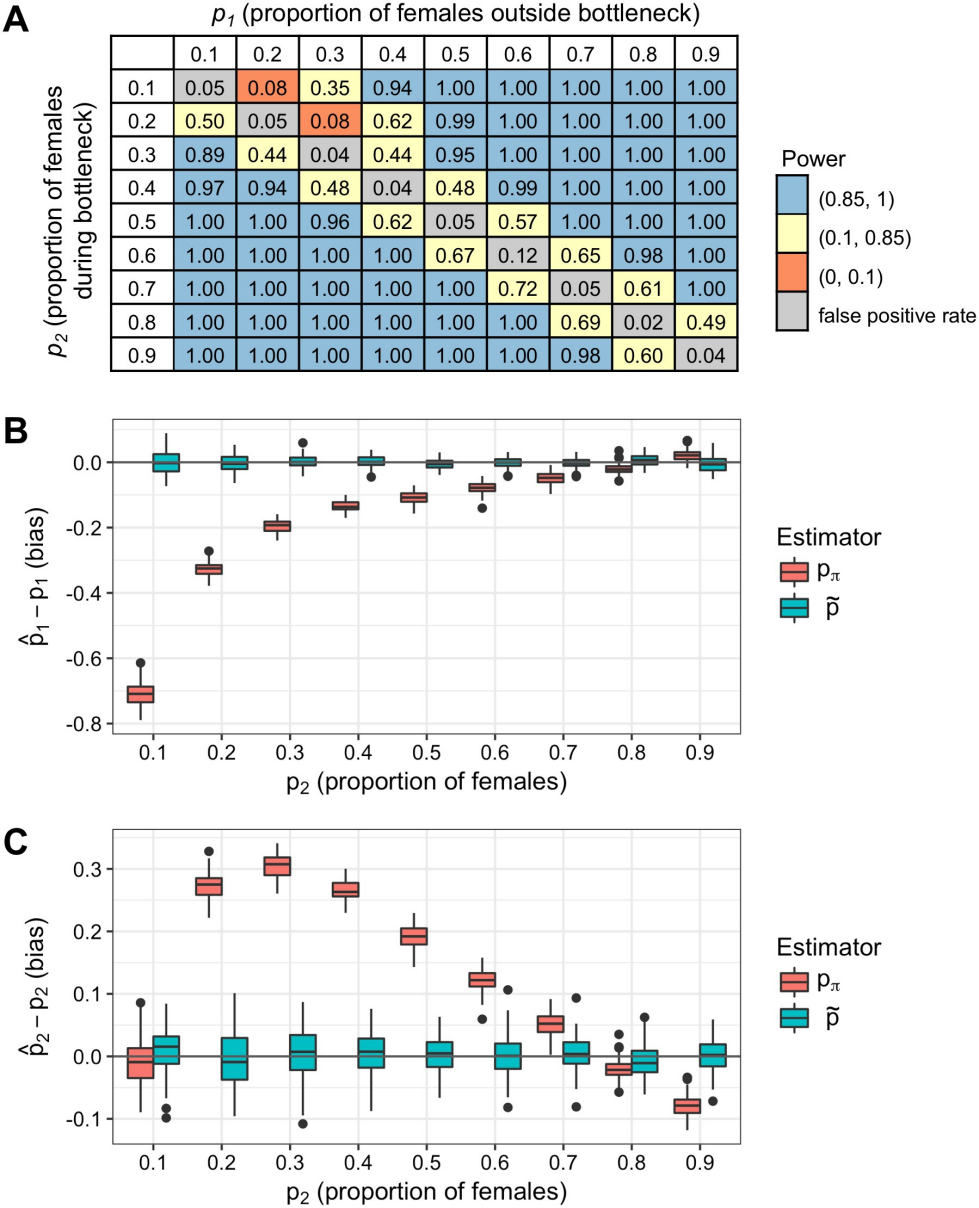
Simulated bottleneck with changing sex-bias. Test for changing sex-bias applied to bottleneck simulations with a proportion of females of *p*_1_ outside the bottleneck and *p*_2_ during the bottleneck. (A) Off-diagonal entries are power: values greater than 0.85 are in blue, values between 0.85 and 0.10 are in light yellow, and values less than 0.10 are in orange. Entries on the diagonal are false positive rates and are in gray. (B, C) Data was simulated with a female bias outside the bottleneck (*p*_1_ = 0.8) and a varying proportion of females during the bottleneck, *p*_2_. Our method produces separate estimates for *p*_1_ and *p*_2_ ($\tilde{p}$, in blue), while the the *π*-based estimator produces one estimate (*p*_*π*_, in red). Bias of estimators of the proportion of females (B) outside the bottleneck, $\hat{p}-p_{1}$, and (C) during the bottleneck, $\hat{p}-p_{2}$, is shown for both methods. The *π*-based estimator is biased when *p*_1_ and *p*_2_ differ (here, when *p*_2_ is not 0.8), whereas our estimator is unbiased.

We next simulated a bottleneck with a changing sex-bias, where the proportion of females is *p*_1_ outside the bottleneck (i.e., before and after the bottleneck) and *p*_2_ during the bottleneck. We applied the test for changing sex-bias to this data and report power in the off-diagonal entries of Fig 7A; false positive rates are on the diagonal, where sex-bias is constant. The test has power of 1.0 when the values of *p*_1_ and *p*_2_ differ by at least 0.2 on the grid of *p* values, which are 0.1 apart. The distribution of the test statistic Λ_1_ is shown in S5 Fig. A single statistic such as *Q*_*π*_ cannot discriminate between a constant sex-bias and one that is changing over time. This is demonstrated by the simulation with an overall female bias (*p*_1_ = 0.8) and a male-biased bottleneck (*p*_2_ = 0.2) in S6 Fig: the estimator *p*_*π*_ based on *Q*_*π*_ is 0.473, which corresponds to slight male bias overall; in contrast, our method recovers the true values of *p*_1_ and *p*_2_ corresponding to a sex-biased bottleneck. Sex-bias estimates for simulations with *p*_1_ = 0.8 with all values of *p*_2_ are shown in Fig 7B and 7C: while our estimator $\tilde{p}$ is unbiased and recovers the true parameters *p*_1_ and *p*_2_, *p*_*π*_ is intermediate between the two values, so its bias varies with *p*_2_. Estimates of the proportion of females for both methods and for all values of *p*_1_, *p*_2_, and *p*_3_ are in S1 Table. For strongly male-biased bottlenecks where *p*_2_ is 0.1 or 0.2, *Q*_*π*_ estimates are downwardly biased. For example, a simulated female bias of *p*_1_ = 0.6 and male-biased bottleneck of *p*_2_ = 0.1 has an estimated *Q*_*π*_ of 0.505, which corresponds to a nonsensical *p*_*π*_ of -0.226, while our estimator recovers the true values of *p*. This highlights the importance of estimating sex-bias in the context of a demographic history.

#### Comparison to KimTree

To compare the performance of our method to a recent sex-bias inference method that uses data from multiple populations, KimTree [23], we applied both methods to multi-population data simulated with sex-biased bottlenecks (S10A Fig). KimTree estimates the one value of the effective sex ratio (ESR), which we refer to as the proportion of females *p*, per population branch. To estimate the ESR prior to a population split, KimTree requires data from an outgroup, so we simulated a third population to estimate the ESR for branch 4. All three populations were simulated with a pervasive female bias (*p*_1_ = 0.8) and a male-biased bottleneck (*p*_2_ = 0.2) with parameters similar to those used in the “Population bottleneck” simulations above: a male-biased bottleneck occurs on branch 4 before populations 1 and 2 split, which affects both of those populations, and another occurs on branch 3, which only affects population 3. Applied to this data, KimTree estimates a female bias for all branches and does not detect the changing sex-bias on branches 3 or 4 (S10B Fig). This could be because KimTree estimates only one ESR per branch or because it does not model population size changes. We next applied our method with a bottleneck demographic model to each marginalized, single-population site frequency spectrum. Our method, which produces one ESR estimate per epoch per population, correctly estimates a male bias during the bottleneck and a female bias outside the bottleneck in all three populations (S10C Fig). In addition, our method is much faster than KimTree: per simulation replicate, KimTree took approximately 31 hours with 6 parallel threads, whereas our method took approximately 10.4 minutes with a single thread, making our method approximately 175 times faster if multi-threading is ignored.

### Applications to sequence data

#### 1000 Genomes Project exomes

To estimate sex-bias in human populations, we first applied our method to high-coverage exome data from the 1000 Genomes Project (approximately 30x coverage). We restricted our analysis to synonymous sites as in other studies of human demographic histories from exome data [27, 28]. Autosomal and X-chromosomal SFS for a European population (CEU) and the African Yoruban population (YRI) are shown in S7 Fig. Europeans (S7A Fig) have an excess of rare X-chromosomal variants relative to the autosomes, which is expected given recent rapid growth in Europeans, while Yorubans have a slight relative excess of rare autosomal variants (S7B Fig). The ratio of mutation rates *α* = *μ*_*M*_/*μ*_*F*_ is a free parameter in our model, so we perform a grid search over *α* to maximize the joint autosomal-and-X-chromosomal likelihood (Eq 12). The maximum likelihood value of *α* is 3, in agreement with a previous estimate from human pedigree data [29]. The proportion of females estimated by our method, $\tilde{p}$, for Europeans and Yorubans fit with different demographic models are in Table 1. Yorubans fit with a misspecified constant population size have an estimated $\tilde{p}$ which is 0.359. A more realistic two-epoch model with old growth (a 2.02x expansion 221 thousand years ago) has a higher likelihood and gives a $\tilde{p}$ of 0.465, corresponding to a male bias. For the European population, using a misspecified constant size model gives a nonsensical $\tilde{p}$ of -0.019 and lower likelihood. A two-epoch model with recent growth (23x expansion 4.7 thousand years ago) has a higher likelihood and gives a $\tilde{p}$ of 0.080. A more realistic model, consisting of a 0.93x bottleneck 51 thousand years ago followed by exponential growth that increased the population’s size by 51x [26], has the highest likelihood and gives a $\tilde{p}$ of 0.435. In both populations, the best-fitting models are male-biased: $\tilde{p}=0.465$ for Yorbans with an old growth model and $\tilde{p}=0.435$ for Europeans with a bottleneck followed by recent exponential growth.

**Table 1:** Sex-bias estimates from 1000 Genomes Project exome data. Our method run with the specified demographic models for Yorubans (YRI) and Europeans (CEU) gives estimates of the proportion of females, $\tilde{p}$, in the last column. The best-fitting models (“Growth” for YRI, “Bottlegrowth” for CEU) are consistent with a slight male bias. Constant models do not estimate any population size change parameters as denoted by “N/A”, and “kya” stands for “thousands of years”.

Population	Model	Population size change parameter estimates	$\tilde{p}$
YRI	Constant	N/A	0.359
YRI	Growth	2.02x expansion 221 kya	0.465
CEU	Constant	N/A	-0.019
CEU	Growth	23x expansion 4.7 kya	0.080
CEU	Bottlegrowth	0.93x bottleneck 51 kya, 51x exponential growth starting 5.1 kya	0.435

#### 1000 Genomes Project whole genomes

**Fig 8:**
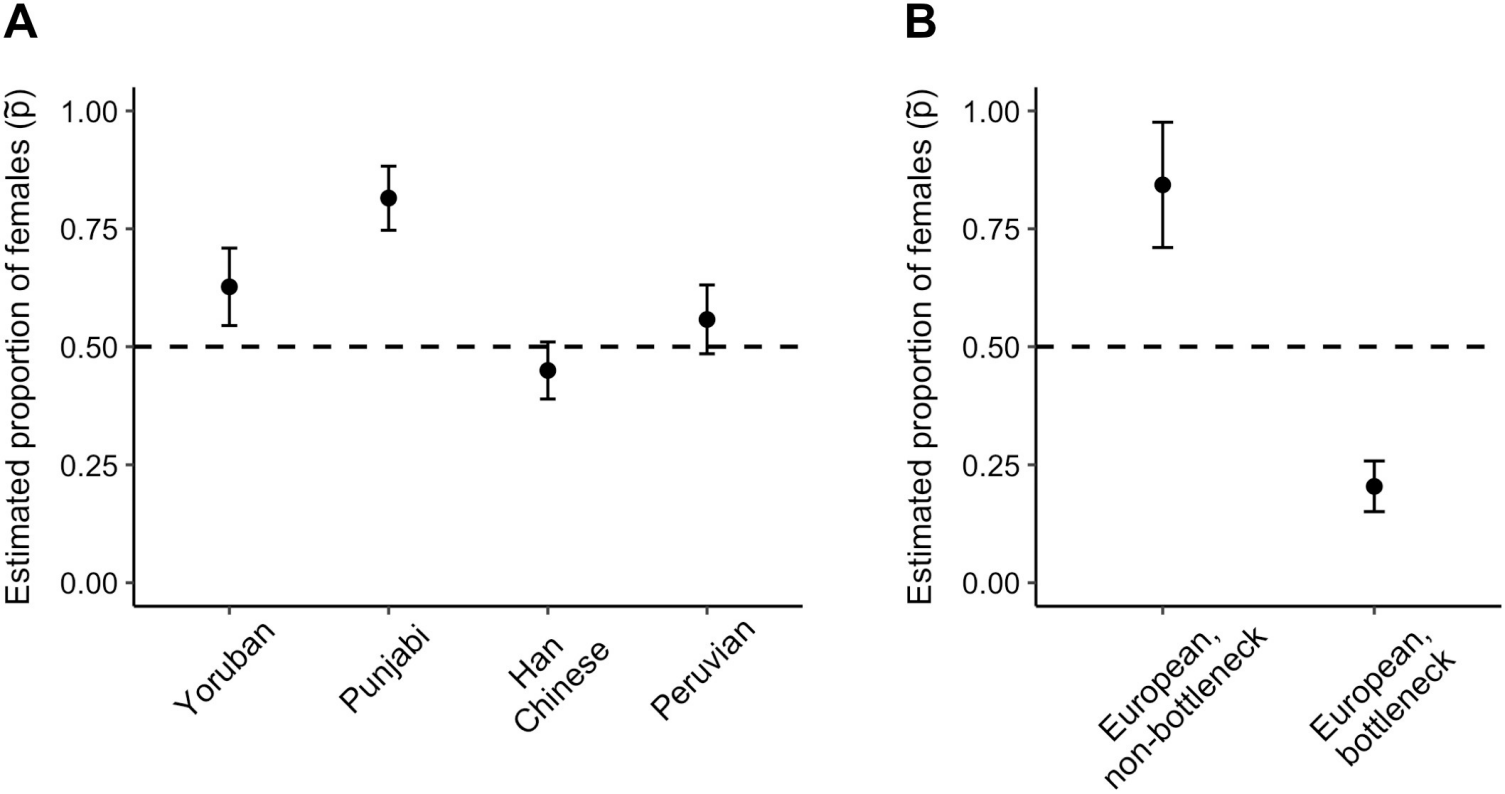
Sex-bias estimates from 1000 Genomes Project high-coverage whole genomes. Estimates of and 95% confidence intervals for the proportion of females from our method for the best-fitting demographic model for each population. Demographic models are defined in the main text. (A) Our method infers a constant proportion of females, $\tilde{p}$, for Yorubans (best demographic model: old growth), Punjabis (complex model), Southern Han Chinese (complex model), and Peruvians (bottleneck model). (B) Our method infers a changing proportion of females for Europeans (complex model) with a female bias outside bottleneck ($\tilde{p_{1}}$, “non-bottleneck”) and a male bias during the bottleneck ($\tilde{p_{2}}$, “bottleneck”).

We next analyzed 159 unrelated females from five populations from Phase 3 of the 1000 Genomes Project who were sequenced to high coverage on the Complete Genomics platform [24]. We restricted our analysis to non-coding regions of chromosomes 7 and X, which are approximately the same physical size (∼150MB) [30]. To filter this data, we removed regions that might have been subject to natural selection or are prone to sequencing error (for details, see Materials and methods). We also removed regions closer than 0.2cM to the nearest gene to reduce differential strengths of background selection, the effect of purifying selection on linked loci, on the X chromosome and autosomes as in [19]. On this filtered data, we fit single-population extrapolations of demographic models used in previous studies of these populations [27, 28]. To reduce the impact of linkage on our inference (specifically, the differential linkage on the autosomes and the X chromosome), we used a conventional bootstrap to estimate standard errors of parameters. Full likelihood ratio testing outcomes are in S2 Table, and the best-fitting model results are shown in Fig 8, which are based on the underlying data in S3 Table.

Our method estimates a constant sex-bias (i.e., $\tilde{p}$ differs from 0.5) for all populations except Europeans (Fig 8A). The best-fitting model for Yorubans (YRI) with older growth and a small amount of recent growth (“Old growth”) has a constant female bias of $\tilde{p}=0.63$. The best-fitting model for Punjabis from Lahore (PJL) is the complex model involving an older expansion, a bottleneck during the Out-of-Africa migration, and recent exponential growth (“complex”), and has a female bias ($\tilde{p}=0.815$). The Southern Han Chinese (CHS) are also best fit by the complex model and are inferred to have a male bias ($\tilde{p}=0.450$), which is consistent with the previous observation of more drift on the X chromosome relative to the autosomes in East Asians than in Europeans [31]. Peruvians (PEL) have a slight overall female bias for the best-fitting bottleneck model (“bottleneck”), which might capture recent sex-biased admixture. We note that our estimated confidence intervals are conservative: although the LRT result for Southern Han Chinese and Peruvians is a constant sex-bias where *p* differs from 0.5, the confidence intervals for *p* for these populations overlap 0.5 slightly. The best-fitting model for Europeans (CEU) is the complex model (S8 Fig) with a changing sex-bias (Fig 8B): our method infers a male-biased bottleneck during the Out-of-Africa migration ($\tilde{p_{2}}=0.204$) and a female bias outside the bottleneck ($\tilde{p_{1}}=0.843$). The exome results from Yorubans and Europeans do not have this signal, which could be due to the differential strengths of background selection on the X chromosome and autosomes [12]. Since purifying selection is stronger on the X chromosome, it decreases genetic diversity more on the X chromosome than on the autosomes and reduces $N_{e}^{X}$ more than $N_{e}^{A}$; indeed, the estimated proportion of females is lower from exome data than from whole-genome sequencing data. Taken together, these results describe a female bias in human populations with a male-biased bottleneck, which is estimated well from non-coding genetic data.

## Discussion

Human sex-bias studies have reached conflicting conclusions due to the type of genomic loci and statistics used [20]. An important confounder is population size change, which can bias sex-bias inferences. To this end, we developed a sex-bias inference method that accounts for demographic history and takes X-chromosomal and autosomal genetic data as input. When applied to coalescent simulations, our method has better power than conventional estimators to estimate an overall sex-bias for arbitrary demographic histories; in addition, our method can detect a changing sex-bias. We also applied our method to human data from the 1000 Genomes Project [24].

There are two main issues with conventional approaches that test for sex-bias with a single summary statistic such as *Q*. The first issue is that the null expectation of *Q* is not 0.75 for a population which has changed in size, so a test comparing *Q* to 0.75 for a population of non-constant size can be underpowered or have false positives [20]. The second issue is that a single summary statistic cannot localize the source of sex-bias to a particular time epoch. For example, for data simulated with a bottleneck and varying amount of sex-bias (S1 Table), a population with no sex-bias (*p* = 0.5) which underwent a female-biased bottleneck (*p* = 0.7) has a *Q* of 0.731, which is similar to the *Q* of 0.737 that a population with a strong female bias (*p* = 0.8) and strongly male-biased bottleneck (*p* = 0.2) has. As a result, these scenarios cannot be distinguished by *Q* alone. Based on simulated data, our test for sex-bias is more powerful than one based on *Q* and is well-powered for demographic events relevant to human history, such as recent expansions and bottlenecks.

Decreasing *p* from 0.5 by some amount, as for a male bias, changes $N_{e}^{X}/N_{e}^{A}$ more than increasing *p* by the same amount, as for a female bias (Eqs 1 and 2). Despite this, our test for a changing sex-bias has good power for all values of *p* on data simulated with a bottleneck. However, a sex-bias estimator that does not account for population size change, such as *p*_*π*_, is more biased when *p* is small (i.e. for a male bias). In bottleneck simulations with a strong male bias, *p*_*π*_ is downwardly biased and at times negative. This is because the strong, recent bottleneck combined with the strong male bias reduces X-chromosomal genetic diversity more than autosomal genetic diversity.

Bottlenecks with a changing proportion of females are relevant to human history, particularly since some bottlenecks correspond to long-range migrations which are hypothesized to have been sex-biased. A bottleneck alone biases conventional sex-bias estimators [17]. Applied to data simulated with a bottleneck under the null of constant sex-bias (*p*_1_ = *p*_2_ = *p*_3_ = 0.5), a conventional estimator is biased and estimates a persistent male bias (*p*_*π*_ = 0.399), whereas our sex-bias estimator is unbiased ($\tilde{p_{1}}=0.503$, $\tilde{p_{2}}=0.496$, $\tilde{p_{3}}=0.500$). Using our method, we find evidence for a male-biased bottleneck out of Africa and have good power to detect such a sex-bias based on simulated data. To our knowledge, this is the first direct test of this hypothesis based on whole-genome sequence data.

A recent method by Clemente et al., KimTree, estimates sex-bias from multi-population data [23]. Our method compliments KimTree in that both offer insight into sex-bias, and each one has a different focus. Our method operates on data from a single population and explicitly models population size changes, while KimTree requires multi-population data and does not explicitly model population size change. Since KimTree estimates one effective sex ratio (i.e., the proportion of females) per branch, it cannot detect sex-bias that changes on a single branch; our method can, and we have shown in simulations that it has good power to do so. Our method does not require an outgroup or knowledge of divergence events, and so can be applied to datasets where multi-population data is not available, including those from ancient samples. In addition, our method is much faster than KimTree: our method ran on a laptop in a few hours with a single thread, whereas KimTree took several days to run, even when multiple threads were used.

Applied to 1000 Genomes Project whole-genome sequence data, our method infers a pervasive female bias in globally-distributed populations. This is consistent with human anthropological literature, which suggests that males have a greater variance in reproductive success than females [5]. In addition, our method identifies a male-biased bottleneck out of Africa based on data from Europeans; the lack of this signal in the other non-African populations may be due to insufficient sample sizes or misspecified demographic models. Finally, our method infers a male bias in the Southern Han Chinese, which is consistent with previous observations. Our results are generally in agreement with those from KimTree, which found either a female bias or no bias in most human populations, and a male bias on the branch ancestral to Europeans and Asians [23]. From filtered, putatively neutral whole-genome sequence data far from genes, our method infers a more extreme female bias than Clemente et al., possibly because their estimates are downwardly biased by their inclusion of genic regions, their inability to account for sex-bias that changes along a population branch, or their assumption of a constant population size.

To assess whether sex-bias estimation from exome data is appropriate, we analyzed synonymous sites as in previous studies [26–28]. We used a range of demographic models and obtained estimates of the proportion of females ranging from negative values to nearly 0.5. For the best-fitting demographic models, $\tilde{p}$ is 0.465 for Yorubans and 0.435 for Europeans, similar to previously-obtained *π*-based estimates from non-genic sites closest to genes [19]. Our results also agree with those from another exome study [12] even though it only assessed three values of *p*, and we assessed the full range of *p*. Then, sex-bias inference from exomes is most likely confounded by background selection.

We make some assumptions in our framework. We use an average mutation rate for the autosomes, *μ*_*A*_, and an average rate for the X chromosome, *μ*_*X*_. Though the mutation rate varies across the genome, we use a single SFS for each type of locus, autosomal and X-chromosomal, so mutation rate differences are averaged in the scaled mutation rate parameter, *θ*. This SFS and *θ* are used together to estimate demographic parameters, as is standard in demographic inference literature. In addition, though we do not require that SNPs be thinned to remove linkage disequilibrium before estimating sex-bias from genomic data, we recommend estimating parameter standard errors with a conventional bootstrap, as commonly done in demographic inference [27].

Our implementation of the sex-bias method we developed uses the program ∂a∂i [27], and any demographic inference program that calculates likelihoods will work (e.g. fastsimcoal [32]). Our method could be extended to test for sex-biased admixture or to analyze multiple populations simultaneously, which would expand its utility. In addition, although we only consider common variation (minor allele frequency > 0.05) from which our method has good power to detect older sex-bias, if high-confidence rare variant calls are available, our method could be used to infer more recent sex-bias. This work underscores the importance of modeling demographic history when estimating sex-bias, and our results give new insight into sex-bias in human populations. Our method can infer sex-bias in any sexual population and provides better null models for selection scans than competing methods, producing a more accurate view of population histories.

## Materials and methods

### Accounting for unequal male and female mutation rates

To allow for unequal male and female mutation rates in our framework, we assume a constant female per-site mutation rate, *μ*_*f*_, and a constant male per-site mutation rate, *μ*_*m*_, with ratio given by *α*. For a given value of *μ*_*A*_ and *α*, we obtain the value *μ*_*X*_ as in [33] and used in [18]:

$$\begin{array}{c} \mu_{X}=\mu_{A}\times \frac{2\times (2+\alpha )}{3\times (1+\alpha )} \end{array}$$ (15)

In humans, *α* is greater than 1, which corresponds to a male mutation bias [34]. These values of *μ*_*A*_ and *μ*_*X*_ can be substituted into Eqs 11, 13 and 14.

### A novel sex-bias inference method

We developed a novel method to estimate sex-bias from genetic data and that uses custom demographic functions written in the python programming language. Our method first estimates autosomal parameters then optimizes X-chromosomal parameters, some of which are constrained by the autosomal parameter estimates (see S1 Text, “Likelihood ratio tests for sex-bias: general form”). To estimate demographic parameters, we use the program ∂a∂i, which uses a diffusion approximation to the one-locus, two-allele Wright-Fisher process [27]. To estimate parameters from simulated data, we used the “log_fmin” function in ∂a∂i, which uses the Nelder-Mead optimizer. For both simulated and genetic data, if parameter bounds are hit, we re-start the optimizer from a randomly perturbed point. To estimate parameters from the 1000 Genomes Project data, we perform a grid search over parameters, start ∂a∂i’s optimizer from the grid search optimum, and take the best point as the maximum likelihood point. For the complex demographic models used in the 1000 Genomes Project whole-genome data analysis, we fixed the parameter values of an older African growth event and the time of the Out-of-Africa bottleneck [28] and optimized more recent events. For samples of more than 20 individuals, we use a fine grid (“minGrid” = 150) and a smaller ∂a∂i timescale of 10^−4^ to improve model fitting (S9 Fig). To construct parametric bootstrap confidence intervals, the following procedure is repeated 100 times. A bootstrap sample is simulated with the coalescent simulation program ms [35] using the demographic model, estimated parameters, and linkage structure of the original dataset. We then estimated demographic parameters with ∂a∂i. For each parameter, the 95% confidence interval is estimated as the range of the central 95% of bootstrap samples for that parameter. In the case of $\tilde{p}$, a bootstrap sample is generated based on autosomal and X-chromosomal data.

### Simulating a population of constant size

We first simulated data from independent sites from 1000 unlinked regions that are 5kb in length. To do so, we drew the number of segregating sites for the autosomes and X chromosome as a Poisson random variable with mean parameter given by Eqs 8 and 9, respectively. We first simulated data under the null hypothesis (*p* = 0.5) and calculated the estimators $\tilde{p}$ and $\tilde{\theta }$ with Eqs 6, 13 and 14 as well as the likelihood ratio test statistic Λ for each simulated set of autosomal and X-chromosomal data. We used the distribution of Λ to obtain the empirical critical value of c* = 3.787. We then simulated data under the alternative hypothesis for *p* ranging from 0.2 to 0.8 in steps of 0.1, and calculated power with respect to c*.

We next simulated partially linked sites with ms. We simulated 10,000 independent samples of a 5KB locus in 10 males and 10 females using a per-site mutation rate of 0.001 and a per-site recombination rate of 0.001. Assuming an ancestral population size *N*_*e*_ = 10^4^, we calculated the population size-scaled mutation rate *θ* and the population size-scaled recombination rate *ρ* based on the proportion of females *p*:

$$\begin{array}{c} \theta^{A}=f_{A}\left(p\right)\theta =4p\left(1-p\right)\theta \\ \theta^{X}=f_{X}\left(p\right)\theta =\frac{9p(1-p)}{2(2-p)}\theta \\ \rho^{A}=f_{A}\left(p\right)\rho =4p\left(1-p\right)\rho \\ \rho^{X}=f_{X}\left(p\right)\times \left(2p\right)/\left(1+p\right)\rho =\frac{9p(1-p)}{2(2-p)}\frac{2p}{1+p} \end{array}$$

Autosomal and X-chromosomal data were simulated separately for *p* ranging from 0.2 to 0.8 in steps of 0.1; commands are in S1 Text, “Simulation Commands: Population of constant size”. We formed datasets of two different sizes, 5kb and 50kb, by combining simulated loci. The values $\tilde{p}$, $\tilde{\theta }$, Λ, and critical value c* were calculated analogously to those for simulated independent sites.

We compare the power of our LRT to a test based on *Q*. We calculated $\hat{Q}$ as ${\hat{\theta }}_{X}/{\hat{\theta }}_{A}$ and estimated confidence intervals with a bootstrap. For partially linked sites simulated in ms, $\hat{Q}$ is calculated as ${\hat{\pi }}_{X}/{\hat{\pi }}_{A}$, and confidence intervals are calculate using a bootstrap over independent iterations.

### Simulating population expansion

We simulated a population of which underwent an instantaneous ten-fold expansion 100 generations ago with ms. We simulated a sample of 40 individuals with mutation rate of 1.5 × 10^−8^ per site. As in the simulations for a population of constant size, the X chromosome per-site mutation rate and recombination rate are functions of *p*, the proportion of females. For each *p* ranging from 0.2 to 0.8 in steps of 0.1, we simulated datasets of 5kb and generated 10,000 independent datasets. We made X-chromosomal and autosomal site frequency spectrum and performed likelihood ratio tests (see Results, “Sex-bias tests for a two-epoch model”) for each dataset.

### Simulating population bottlenecks

We simulated a bottleneck with the parameters estimated from European genetic data [26]. The population starts at size 14,500 at 5840 generations ago, experiences a bottleneck to 1861 individuals lasting from 2040 to 920 generations ago, then expands to its final size of 100,000. We simulated sex-bias during epochs by setting effective sizes of X chromosomes and autosomes as per Eqs 1 and 2. The per-site mutation rate is 1.5 × 10^−8^, the locus length is 100Kb, and 50 females are simulated by sampling 100 X chromosomes and 100 autosomes. We averaged 10^5^ independent ms simulation iterations to construct the autosomal and the X-chromosomal site frequency spectrum. We simulated the same proportion of females before and after the bottleneck. We tested for sex-bias with the likelihood ratio framework for a bottleneck (see S1 Text, “Likelihood ratio tests for sex-bias: bottleneck model”).

### Simulation for comparison to KimTree method

We simulated data with ms for three populations with a female bias (*p*_1_ = 0.8). After population 3 splits off, the population ancestral to population 1 and 2 experiences a male-biased bottleneck (*p*_2_ = 0.2) on branch 4, as does population 3 on branch 3 (S10A Fig). We used the same bottleneck parameters (magnitude and times) as in “Simulating population bottlenecks” above. We sampled 100 autosomes and X chromosomes from 50 diploid females per population and performed 100 replicate simulations. We estimated the estimated sex ratio (ESR) for each branch with KimTree [23] and used the program arguments recommend in the manuscript and program documentation: -npilot 20 -lpilot 500 -burnin 10000 -length 20000 -thin 20. We applied our method with a bottleneck model to each marginal frequency spectrum of populations 1, 2, and 3. KimTree was run multi-threaded (6 threads) and our method was run with a single thread.

### Mutation rate parameters used in analysis of human data

Since the male germline per-site mutation rate is higher than the female rate [12], X-chromosomal and autosomal per-site mutation rates differ. In the 1000 Genomes Project exome analysis, we estimate *α* = *μ*_*M*_/*μ*_*F*_ via a grid search. In the 1000 Genomes Project whole-genome data analysis, we assume a value of 3 for *α* (close to the empirical value of 3.6 from [34]), which corresponds to an X-chromosomal to autosomal mutation rate ratio of 5/6 (Eq 15). When estimating *α* via a grid search, *θ*_*X*_ is a free parameter in the X-chromosomal optimization and we perform a grid search to obtain the value of *θ*_*X*_ that results in the best overall likelihood and the optimal value of *α* for the dataset. When assuming an *α* value of 3, it is used to constrain X-chromosomal parameters based on autosomal parameters: we use an autosomal per-site mutation rate of 1.2 × 10^−8^ [29] and divide it by the value of $E[N_{e}^{X}/N_{e}^{A}]$. Then, the X-chromosomal model is optimized using the ∂a∂i Poisson model where *θ* is a fixed input parameter.

### 1000 Genomes Project exome data

We analyzed males and females from the 1000 Genomes Project exome pilot data (2012-03-17 release date). We annotated exome variant calls with SNPeff [36] and kept only synonymous variants. We analyzed chromosome X and chromosome 22, each of which has approximately 3000 segregating sites in the exome targeted sequencing study. We constructed folded site frequency spectra for the European (CEU) and Yoruban (YRI) population samples. The chromosome 22 SFS has a higher dimension than the chromosome X SFS for both populations because the samples contain males and females. As a result, we projected the chromosome 22 SFS down to the dimension of the chromosome X SFS using the hypergeometric projection [27] for visual comparison and analysis.

### 1000 Genomes Project whole-genome data

We downloaded the VCF file from the 1000 Genomes Project FTP site for Complete Genomics SNP calls (release date 2013-08-08) for 159 females from the following five populations: Yorubans (YRI), Punjabis (PJL), Southern Han Chinese (CHS), Peruvians (PEL), and Europeans (CEU). We restricted our analysis to females to control for any differences in assembly and variant calling between males and females. Of the six individuals sequenced based on two cell types (blood and LCL), and we kept calls from one cell type. We used VCFTools [37] version v0.1.13 to remove multi-allelic SNPs and retain biallelic SNPs with quality VQHIGH. We used to plink [38] to set Complete Genomics half-calls to missing and remove the X chromosome pseudo-autosomal regions.

We excluded sites with more than 5% missing genotypes. Sites were filtered as in “Filtering 1000 Genomes Project whole-genome data” below and used to construct autosomal and the X-chromosomal site frequency spectra. The length of each locus is defined as the number of bases where a confident call is made (reference, variant, etc.) which was not removed by the filters described earlier. The locus length is used to convert from time in genetic (i.e., coalescent) units to time in generations and to calculate per-base statistics. To adjust the callable length for SNPs removed during filtering, we multiplied the locus length by the ratio of remaining SNPs to original SNPs. For SFS projected down with a hypergeometric projection, the locus length was similarly adjusted by multiplying by the ratio of SNPs in the projected SFS to the number of SNPs in the original SFS. We do not thin SNPs to remove linkage disequilibrium because the expected values of the SFS are the same for independent sites and for partially linked sites, so demographic point estimates are not affected [27]. Confidence intervals were constructed with standard errors estimated from a conventional bootstrap of 1MB blocks across 100 iterations. We used the average per-site mutation rate of 1.5 × 10^−8^.

### Filtering 1000 Genomes Project whole-genome data

For analyses described in “1000 Genomes Project whole-genome data” above, we stratified variants by their genetic distance to the closest gene in centimorgans (cM) by using closestBed [39] to get the closest gene boundary to each SNP in physical units (basepairs, bp). We then used a linear interpolation on the HapMap sex-averaged recombination map to convert SNP and gene boundary positions to genetic units (cM), and took their difference as the distance of the SNP to the closest gene. We restricted attention to SNPs at least 0.2cM from the nearest gene as in [19] because they are expected to be less affected by background selection. We also removed regions which are putatively under selection, prone to sequencing error, or cause differences in local mutation rates which are contained in the following UCSC tracts [30]: phastConsElements46wayPlacental, simpleRepeat, centeromere/telomere, gap, cpgIslandExt, genomicSuperDups, knownGene, selfChain, rnaCluster, intronEst.

### Program availability

The python source code for our sex-bias inference method and its documentation are freely available for download at https://github.com/shailamusharoff/sex-bias-inference/.

## Supporting information

S1 Text Simulation commands and likelihood ratio test for sex-bias. We give example ms simulation commands to generate autosomal and X-chromosomal data from a population which experienced no sex-bias (*p* = 0.5) or a male bias (*p* < 0.5). We describe the general form of the likelihood ratio tests for sex-bias. We also specify all sex-bias models for a bottleneck demographic history and define all parameters, along with their units.
(PDF)

S1 FigConstant population size simulations with no sex-bias.Estimators from our sex-bias inference method applied to data simulated for a population of constant size under the null hypothesis (*p* = 0.5). Parameter estimates across simulations recover the true parameter in red for (A) the proportion of females, $\tilde{p}$ and (B) the scaled mutation rate, *θ*. (C) Test statistics have a critical value in blue corresponding to a false discovery rate of 0.05.(TIF)

S2 FigConstant population size simulations with male sex-bias.Estimators from our sex-bias inference method applied to data simulated from a population of constant size under the alternative hypothesis (*p* = 0.2). Parameter estimates across simulations recover the true parameter in red for (A) the proportion of females, $\tilde{p}$ and (B) the scaled mutation rate, *θ*. (C) Test statistics of true discoveries are beyond the critical value in blue.(TIF)

S3 Fig Recent growth simulations: Small sample size. We simulated a population which underwent recent growth (55x, 205 generations ago) and varied the amount of constant sex-bias for a small sample of 40 chromosomes. Test statistics for our test of (A) constant sex-bias, Λ_0_, and (B) changing sex-bias, Λ_1_, are shown.
(TIF)

S4 Fig Recent growth simulations: Large sample size. We simulated a population which underwent recent growth (55x, 205 generations ago) and varied the amount of constant sex-bias for a large sample of 5000 chromosomes. Test statistics for our test of (A) constant sex-bias, Λ_0_, and (B) changing sex-bias, Λ_1_, are shown.
(TIF)

S5 Fig Test statistics from bottleneck simulations with a changing sex-bias. We simulated a population which experienced a bottleneck and has the same proportion of females before and after the bottleneck. The gray facet labels are the proportion of females outside the bottleneck (*p*_1_) and the x-axis is the proportion of females during the bottleneck (*p*_2_; here “prop2”). Test statistics for a changing sex-bias (Λ_1_, here “lambda.1.2.val”) are shown with the parametric bootstrap critical value as a horizontal gray line.
(TIF)

S6 FigEstimated proportion of females from a male-biased bottleneck.We show sex-bias estimators from simulations with a female bias outside the bottleneck (*p*_1_ = 0.8) and a male bias during the bottleneck (*p*_2_ = 0.2). Our estimator $\tilde{p}$ in green (“SFS”), recovers the true value of (A) *p*_1_ and (B) *p*_2_, denoted by the gray horizontal line. The estimator *p*_*π*_ in red (“*π*”), gives a single biased estimate.(TIF)

S7 Fig 1000 Genomes Project exome site frequency spectra. Folded site frequency spectra (SFS) for 1000 Genomes Project exomes of (A) Europeans (CEU) and (B) Yorubans (YRI). Autosomal SFS (red, “Auto”) were projected down to have the same dimensions as X-chromosomal SFS (blue, “chrX”). The expected SFS based on the standard neutral model is in dark gray (“snm”).
(TIF)

S8 Fig Demographic model log-likelihoods for 1000 Genomes Project Europeans, whole-genome sequence data. Autosomal demographic model log-likelihoods from 1000 Genomes Project European (CEU) whole-genome sequence data. The best-fitting model, a complex model (“three epoch growth”), has the largest log-likelihood.
(TIF)

S9 Fig The ∂a∂i timescale and grid parameters affects recovery of simulation parameters. Log-likelihoods of data simulated under a two-epoch model for a grid of fold-size changes, *ν* (“nu”), on the x-axis and times, *τ* (“tau”), on the y-axis. The true simulation parameter is denoted by a black dot, light blue regions have better log-likelihoods, and dark blue regions have poorer log-likelihoods. (A) With the default ∂a∂i timescale parameter of 1*e*^−3^ and a coarse grid, the true point does not have the best likelihood. (B) With a smaller timescale parameter of 1*e*^−4^ and a finer grid, the true point has the best likelihood.
(TIF)

S10 Fig Comparison of our method to KimTree on data simulated with sex-biased bottlenecks. Data simulated from three populations, each of which experience a sex-biased bottleneck. (A) Multi-population tree model where populations are nodes. The contemporary populations 1, 2, and 3 are sampled. A male-biased bottleneck occurs on branches 3 and 4 where the proportion of females outside the bottleneck, *p*_1_, is 0.8 and the proportion of females during the bottleneck, *p*_2_, is 0.2. The proportion of females is 0.8 on branches 1 and 2. (B) KimTree estimates a female bias on each branch (*ξ*) and does not detect the male-biased bottlenecks because it cannot fit sex-bias parameters that change on a branch. (C) Our method correctly estimates a female bias outside the bottleneck (“p1”) and a male during the bottleneck (“p2”) in all three populations.
(TIF)

S1 Table Estimated proportion of females from bottleneck simulations with a changing sex-bias. Sex-bias estimates from a simulated population that experienced a bottleneck with a proportion of females *p*_1_ before the bottleneck, *p*_2_ during the bottleneck, and *p*_3_ after the bottleneck. True values are in columns 1-3, our estimators are in columns 4-6, and the single *π*-based estimator *p*_*π*_ is in column 7 (“p_pi”). Our method recovers true parameters well whereas *p*_*π*_ is biased by size changes and changing sex-bias.
(XLSX)

S2 Table Likelihood ratio testing outcomes from 1000 Genomes Project whole-genome sequencing data. Test for sex-bias applied to globally-distributed populations. The best-fitting demographic model is shown. For the sex-bias test comparing the X-chromosomal models X0 and X1, the likelihood ratio test statistic and p-value are shown on the line with “X1” in the “X-chromosomal model” column, and analogously for the changing sex-bias test comparing the models X1 and X2. Values that are not estimated by a model are denoted by “-”. For models with a bottleneck (“Complex” and “Bottleneck”), “p1_hat” is the estimated proportion of females outside the bottleneck and “p2_hat” is the estimated proportion of females during the bottleneck. The best-fitting X-chromosomal model based on the nested likelihood ratio tests is in bold.
(XLSX)

S3 Table Sex-bias estimates from 1000 Genomes Project whole-genome sequencing data. Data underlying Fig 8. For each population, estimates, standard errors, and lower and upper bounds of a 95% confidence interval are shown for the estimated proportion of females from the best-fitting model. All populations except Europeans have a single estimate of *p*, and Europeans have an estimate *p*_1_ (“p1”) outside the bottleneck and *p*_2_ (“p2”) during the bottleneck.
(XLSX)
