# Supplementary material for: The inference of sex-biased human demography from whole-genome data
Source: PLoS Genet. 2019 Sep 20;15(9):e1008293. doi: 10.1371/journal.pgen.1008293 (PMC6774570; doi:10.1371/journal.pgen.1008293)
Supplement: S1 Text — We give example ms simulation commands to generate autosomal and X-chromosomal data from a population which experienced no sex-bias (p = 0.5) or a male bias (p < 0.5). We describe the general form of the likelihood ratio tests for sex-bias. We also specify all sex-bias models for a bottleneck demographic history and define all parameters, along with their units. (PDF) [file pgen.1008293.s001.pdf]

Supporting Information: The inference of sex-biased human demography from whole-genome data

Shaila Musharoff, Suyash Shringarpure, Carlos Bustmante, Sohini Ramachandran

Supporting Information

Simulation Commands

Population of constant size We used the program `ms` to simulate data from a population with a sex-biased demographic history. For a population of constant size with no sex-bias ($p = 0.5$) or one with a strong male bias ($p = 0.2$), we used the following commands to simulate autosomal and X-chromosomal data:

```
ms 40 10000 -t 30.0 -r 30.0 5000    # autosomal, p = 0.5
ms 30 10000 -t 22.5 -r 15.0 5000    # X chromosome, p = 0.5
ms 40 10000 -t 19.2 -r 19.2 5000    # autosomal, p = 0.2
ms 30 10000 -t 12.0 -r 4.0 5000     # X chromosome, p = 0.2
```

Analogous commands were used for populations with other demographic histories.

Likelihood ratio tests for sex-bias: general form

We define a demographic history as a set of population sizes $(N_{e1}, N_{e2}, ...N_{eT})$ which go forward in time (i.e., N_{e1} is the ancestral population size) and correspond to a set of $T - 1$ size changes and T epoch durations. The size changes $\vec{\nu} = (\nu_1, \nu_2, ..., \nu_{T-1})$, which occur instantaneously or exponentially, are defined as the size at the end of an epoch relative to the ancestral population size. The epoch durations $\vec{\tau} = (\tau_1, \tau_2, ..., \tau_T)$ are in units of genetic time scaled by the ancestral population size. We assume the X chromosome has the same demographic model (i.e. number and kind of size changes) as the autosomes.

During the epoch t, $t = 1 \ldots T$, $N_t^X = c_t \times N_t^A$ for some constant c_t, which is a function of p_t, the female fraction of the effective size during the epoch. To test for sex-bias during epoch i, the constraints $\nu_t^X = c_{i+1}/c_1 \times \nu_t^A$ and $\tau_t^X = 1/c_{i+1} \times \tau_t^A$ are used (see derivation for a bottleneck model in "Likelihood ratio tests for sex-bias: bottleneck model" below). Since we define the population-scaled mutation

rate, θ, in terms of the ancestral population size, it is constrained by c_1 of the first epoch: $N_1^X = c_1 \times N_1^A$ and $\theta^X = c_1 \times \theta^A$.

Likelihood ratio tests for sex-bias: bottleneck model

Immediately following a population bottleneck, heterozygosity declines rapidly it reaches a minimum amount due to the reduced population size and then increases slowly with the influx of new mutations [3]. The bottleneck reduces the mean number of alleles at a locus, mainly by removing singletons and doubletons [2], which shifts the mode of the allele frequency distribution toward more common alleles [1]. Bottlenecks affect the X chromosomes and autosomes differently due to their different effective population sizes: in the generations immediately following the end of a bottleneck when a population has recovered to its pre-bottleneck size, the X chromosome will have lost more genetic diversity than the autosomes, and the ratio of their genetic diversity will be less than 0.75. Some time later, the X chromosome will recover genetic diversity faster than the autosomes, and it is possible for the ratio of their genetic diversity to be greater than 0.75 [4].

A bottleneck demographic model has two more parameters than a single size change model, so there are more likelihood ratio tests for a bottleneck than for the single size change model described in the main manuscript, "Sex-bias tests for a two-epoch model". Using the ∂a∂i conventions, the bottleneck parameters are:

- ν_B: the ratio of the bottleneck and ancient population sizes
- ν_F: the ratio of the contemporary and ancient population sizes
- τ_B: the length of bottleneck (in units of $2 \times N_{ancestral}$ generations)
- τ_F: time since bottleneck recovery (in units of $2 \times N_{ancestral}$ generations)

We first fit the parameters of a bottleneck model to the autosomal data. We then fit the following models for X-chromosomal data with the ∂a∂i Poisson model so that θ_X is an explicit parameter.

Model 0

No sex-bias: $p = 0.5$ for all epochs. A bottleneck model has three epochs indexed by $i = 1, 2, 3$. The equations relating the autosomal and X-chromosomal

parameters are as follows:

$$
\begin{aligned}
N_i^X &= 3/4 * N_i^A \\
\nu_B^X &= N_B^X/N_1^X = (3/4 * N_2^A)/(3/4 * N_1^A) = N_2^A/N_1^A = \nu_B^A \\
\nu_F^X &= \nu_F^A \\
\tau_1^A &= \tau_B/N_1^A \\
\tau_1^X &= \tau_B/N_1^X \\
\tau_B &= \tau_1^A * N_1^A = \tau_1^X * N_1^X \\
\tau_1^X &= (\tau_1^A * N_1^A)/N_1^X = (\tau_1^A * N_1^A)/(3/4 * N_1^A) = 4/3 * \tau_1^A
\end{aligned}
$$

Rearranging the above, the X-chromosomal parameters are related to the autosomal parameters by the constant $c = 3/4$ for each of the size changes, which are indexed by $j = B$ during the bottleneck and $j = F$ after the bottleneck:

$$
\begin{aligned}
\nu_j^X &= \nu_j^A \\
\tau_j^X &= 4/3 * \tau_j^A \\
\theta_1^X &= 3/4 * \theta_1^A
\end{aligned}
$$

Since the X-chromosomal and autosomal fold-size changes are constrained to be the equal, X-chromosomal event times are constrained to be 4/3 the corresponding autosomal times. There are no free parameters in this model, so the likelihood is evaluated without parameter optimization.

Model 1

Constant sex-bias: p is a value other than 0.5 and is the same for all epochs. The X-chromosomal and autosomal fold-size changes are constrained to be equal, and the X-chromosomal event times are constrained to be a constant factor c times the corresponding autosomal times. This constraint parameter is a function of the proportion of females via the reduction factors $f_A(p) = N_e^A/N$ and $f_X(p) = N_e^X/N$:

$$c = f_X(p)/f_A(p)$$

This gives the following constraints:

$$
\begin{aligned}
\nu_j^X &= \nu_j^A \\
\tau_j^X &= 1/c * \tau_j^A \\
\theta_1^X &= c * \theta_1^A
\end{aligned}
$$

Model 2

Sex-biased bottleneck: p is the same before and after the bottleneck, and differs during the bottleneck. ν_F^X and ν_F^A after the bottleneck are the same because the X chromosome and the autosomes undergo the same size change and the proportion of females for the first and last epochs are the same. There are two constraint parameters, c_1 and c_2, which are free parameters:

$$c_1 = f_X(p_1)/f_A(p_1)$$
$$c_2 = f_X(p_2)/f_A(p_2)$$

This gives the following constraints:

$$\nu_B^X = c_2/c_1 * \nu_B^A$$
$$\nu_F^X = \nu_F^A$$
$$\tau_B^X = 1/c_2 * \tau_B^A$$
$$\tau_F^X = 1/c_1 * \tau_F^A$$
$$\theta_1^X = c_1 * \theta_1^A$$

Model 3

Changing sex-bias: p differs for each epoch. There are three constraint parameters, c_1, c_2, c_3, which are free parameters:

$$c_1 = f_X(p_1)/f_A(p_1) \quad (1)$$
$$c_2 = f_X(p_2)/f_A(p_2) \quad (2)$$
$$c_3 = f_X(p_3)/f_A(p_3) \quad (3)$$

This gives the following constraints:

$$\nu_B^X = c_2/c_1 * \nu_B^A \quad (4)$$
$$\nu_F^X = c_3/c_2 * \nu_F^A \quad (5)$$
$$\tau_i^X = 1/c_2 * \tau_i^A \quad (6)$$
$$\tau_i^X = 1/c_3 * \tau_i^A \quad (7)$$
$$\theta_1^X = c_1 * \theta_1^A \quad (8)$$

These models and their corresponding likelihood ratio tests are used to test for sex-bias in a bottleneck model. Analogous models can be defined and used to test for sex-bias in other demographic models.

References

[1] G Luikart, F W Allendorf, and W B Sherwin. Distortion of allele frequency distributions provides a test for recent population bottlenecks. *Journal of Heredity*, pages 238–247, 1998.

[2] Takeo Maruyama and Paul Fuerst. Population bottlenecks and nonequilibrium models in population genetics. II. Number of alleles in a small population that was formed by a recent bottleneck. *Genetics*, pages 675–689, 1985.

[3] Masatoshi Nei, Takeo Maruyama, and Ranajit Chakraborty. The Bottleneck Effect and Genetic Variability in Populations. *Evolution*, 29(1):1–10, 1975.

[4] John E Pool and Rasmus Nielsen. Population size changes reshape genomic patterns of diversity. *Evolution*, 61(12):3001–6, dec 2007.
