# Supplementary figures and images for: The inference of sex-biased human demography from whole-genome data
Source: PLoS Genet. 2019 Sep 20;15(9):e1008293. doi: 10.1371/journal.pgen.1008293 (PMC6774570; doi:10.1371/journal.pgen.1008293)

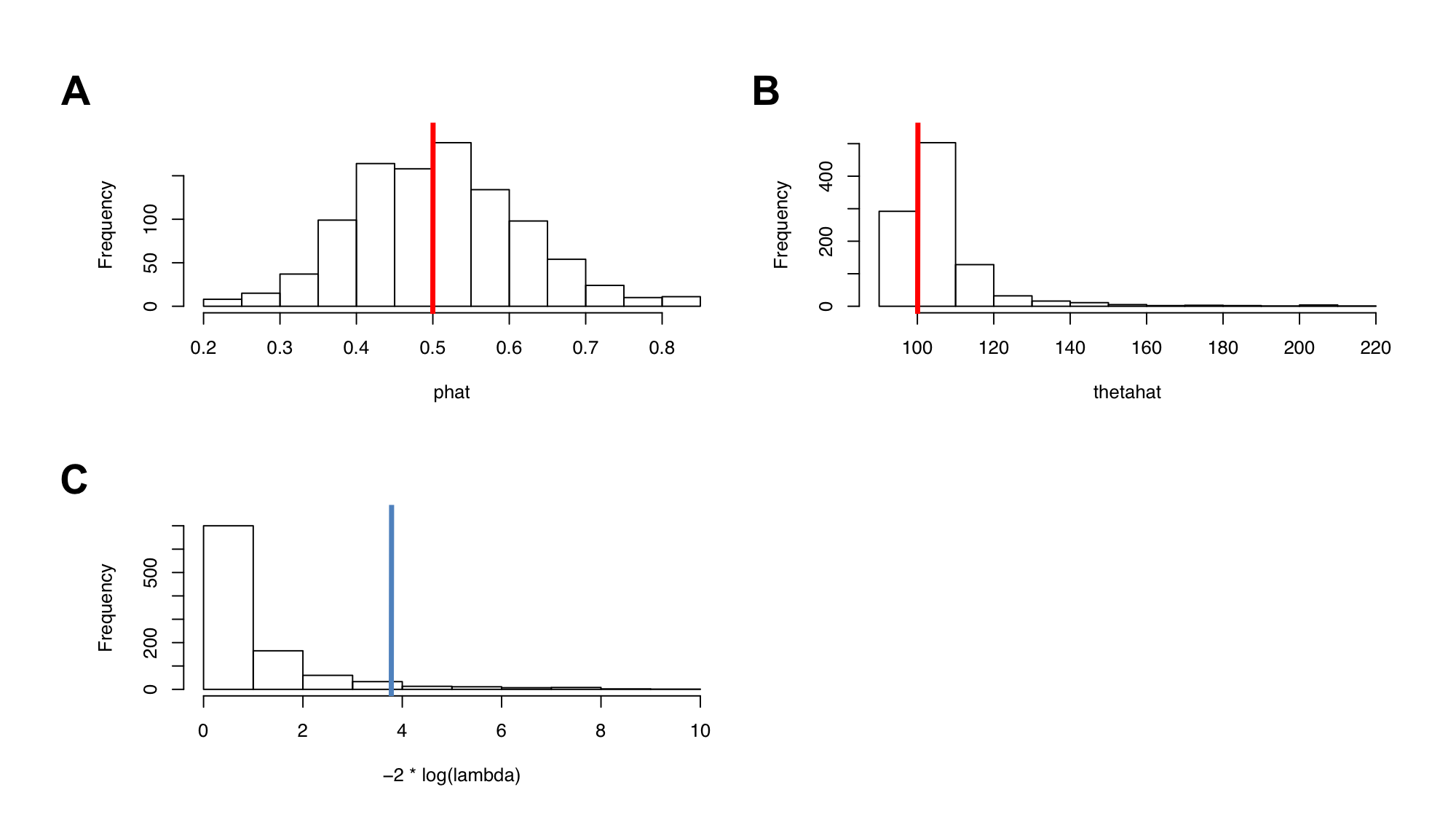

Supplement: S1 Fig — Estimators from our sex-bias inference method applied to data simulated for a population of constant size under the null hypothesis (p = 0.5). Parameter estimates across simulations recover the true parameter in red for (A) the proportion of females, p˜ and (B) the scaled mutation rate, θ. (C) Test statistics have a critical value in blue corresponding to a false discovery rate of 0.05. (TIF) [file pgen.1008293.s002.tif]

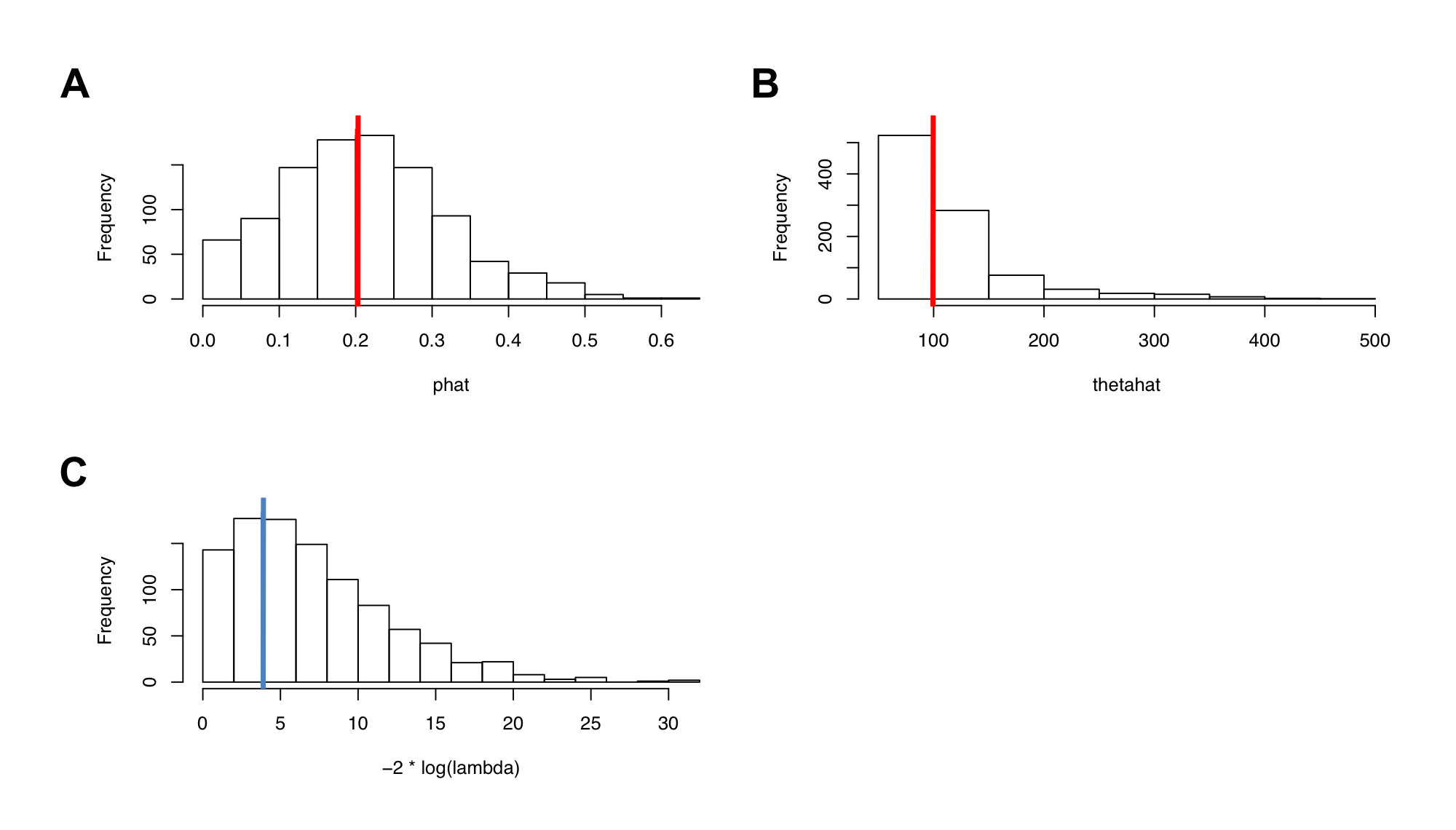

Supplement: S2 Fig — Estimators from our sex-bias inference method applied to data simulated from a population of constant size under the alternative hypothesis (p = 0.2). Parameter estimates across simulations recover the true parameter in red for (A) the proportion of females, p˜ and (B) the scaled mutation rate, θ. (C) Test statistics of true discoveries are beyond the critical value in blue. (TIF) [file pgen.1008293.s003.tif]

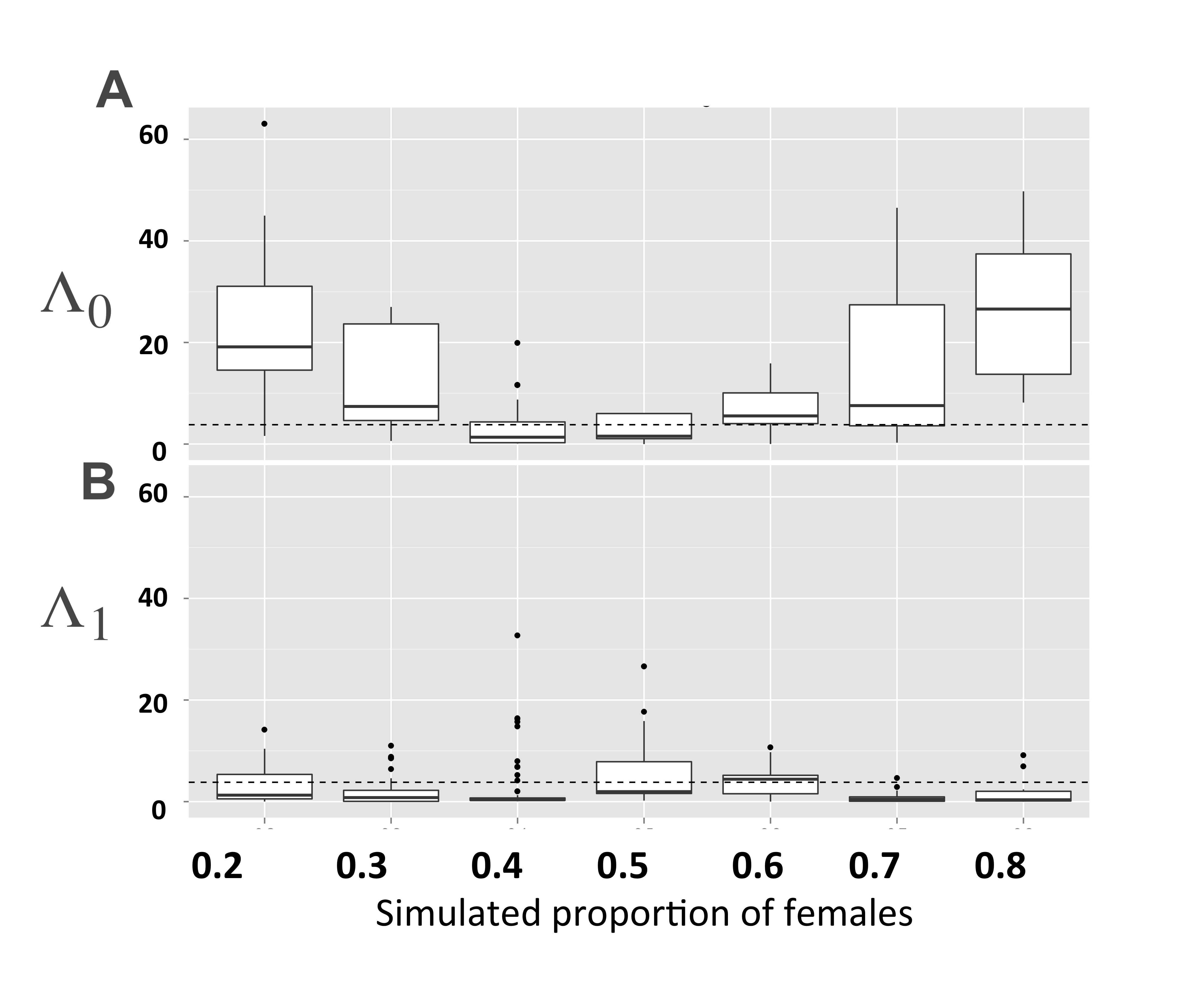

Supplement: S3 Fig — We simulated a population which underwent recent growth (55x, 205 generations ago) and varied the amount of constant sex-bias for a small sample of 40 chromosomes. Test statistics for our test of (A) constant sex-bias, Λ0, and (B) changing sex-bias, Λ1, are shown. (TIF) [file pgen.1008293.s004.tif]

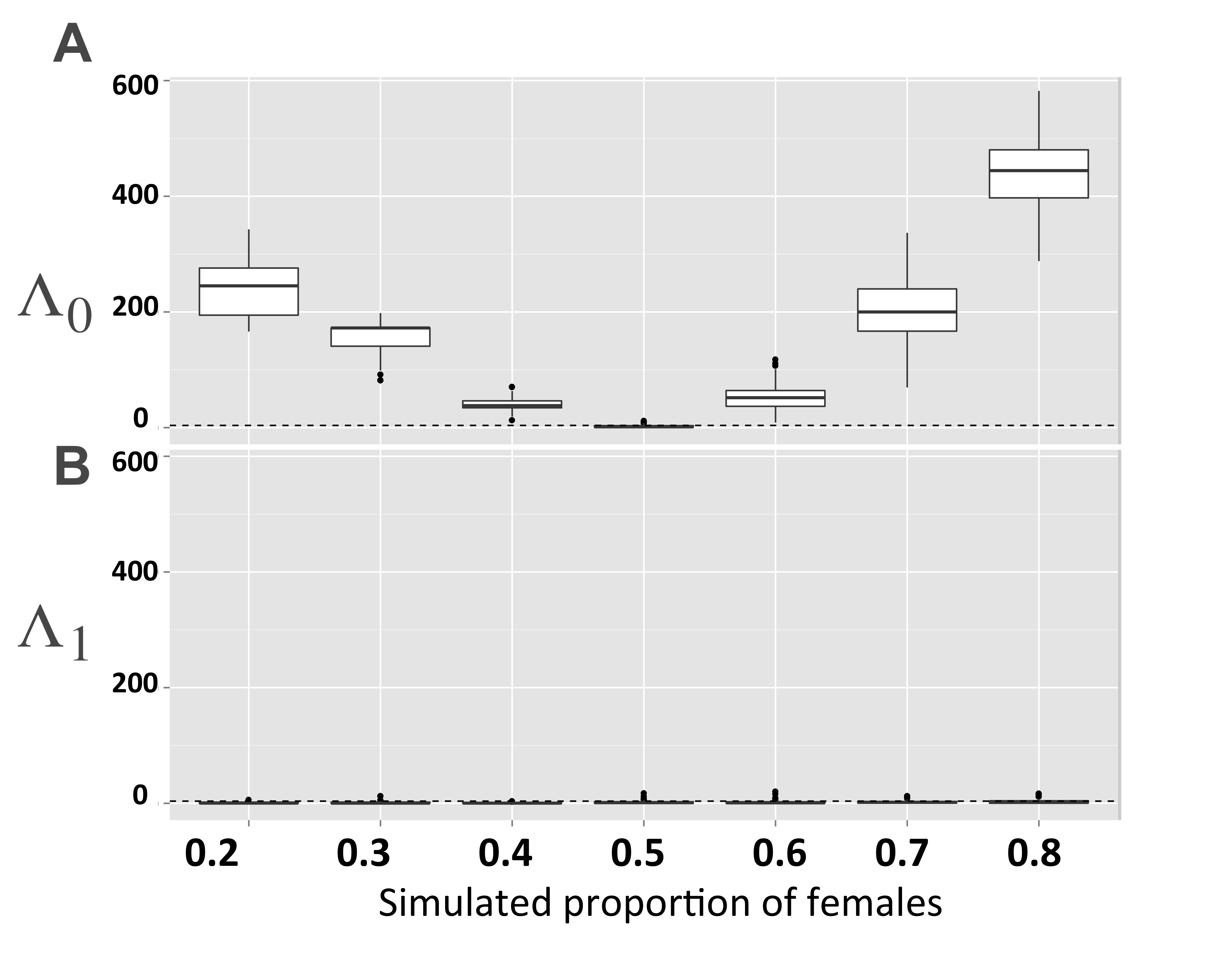

Supplement: S4 Fig — We simulated a population which underwent recent growth (55x, 205 generations ago) and varied the amount of constant sex-bias for a large sample of 5000 chromosomes. Test statistics for our test of (A) constant sex-bias, Λ0, and (B) changing sex-bias, Λ1, are shown. (TIF) [file pgen.1008293.s005.tif]

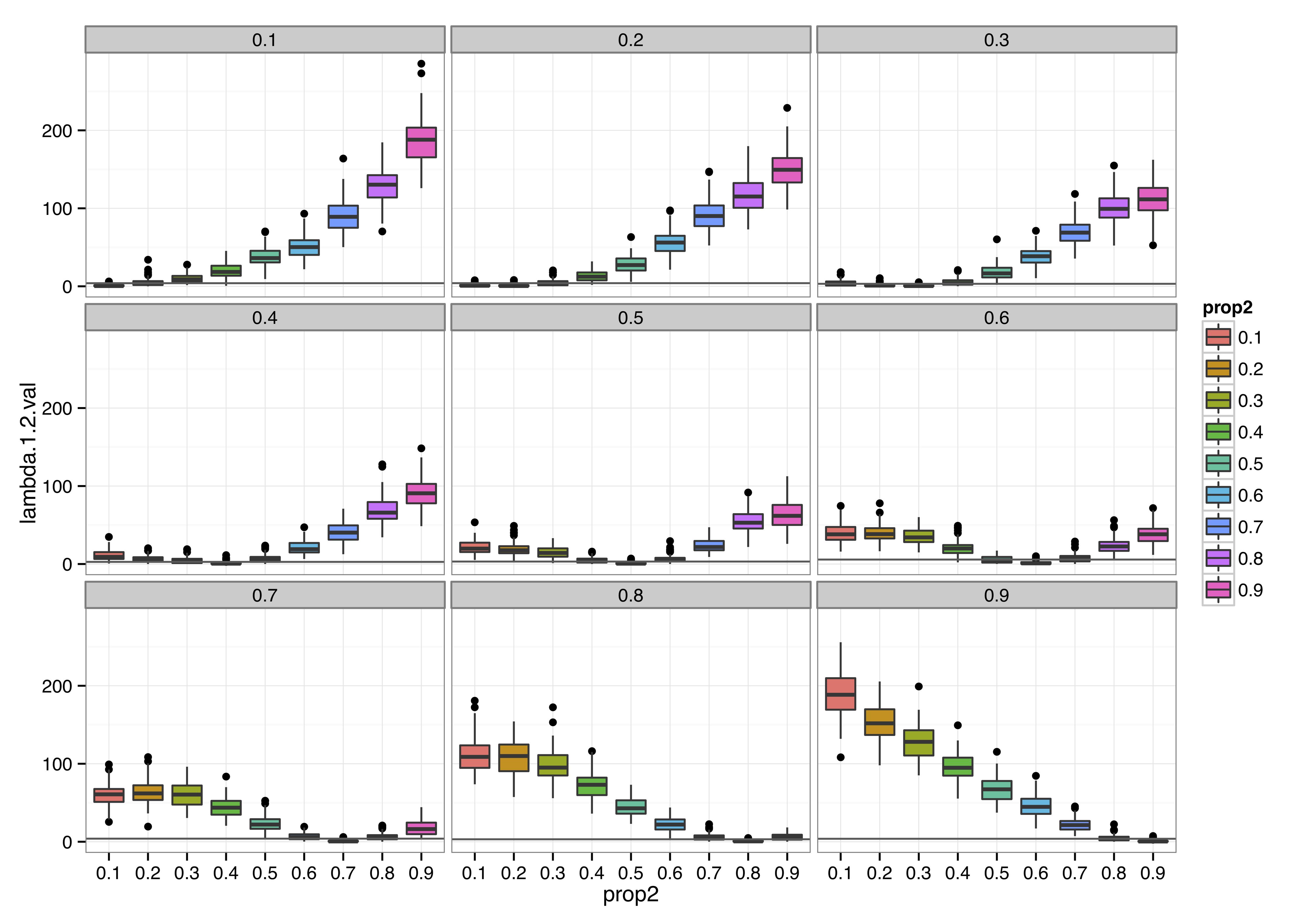

Supplement: S5 Fig — We simulated a population which experienced a bottleneck and has the same proportion of females before and after the bottleneck. The gray facet labels are the proportion of females outside the bottleneck (p1) and the x-axis is the proportion of females during the bottleneck (p2; here “prop2”). Test statistics for a changing sex-bias (Λ1, here “lambda.1.2.val”) are shown with the parametric bootstrap critical value as a horizontal gray line. (TIF) [file pgen.1008293.s006.tif]

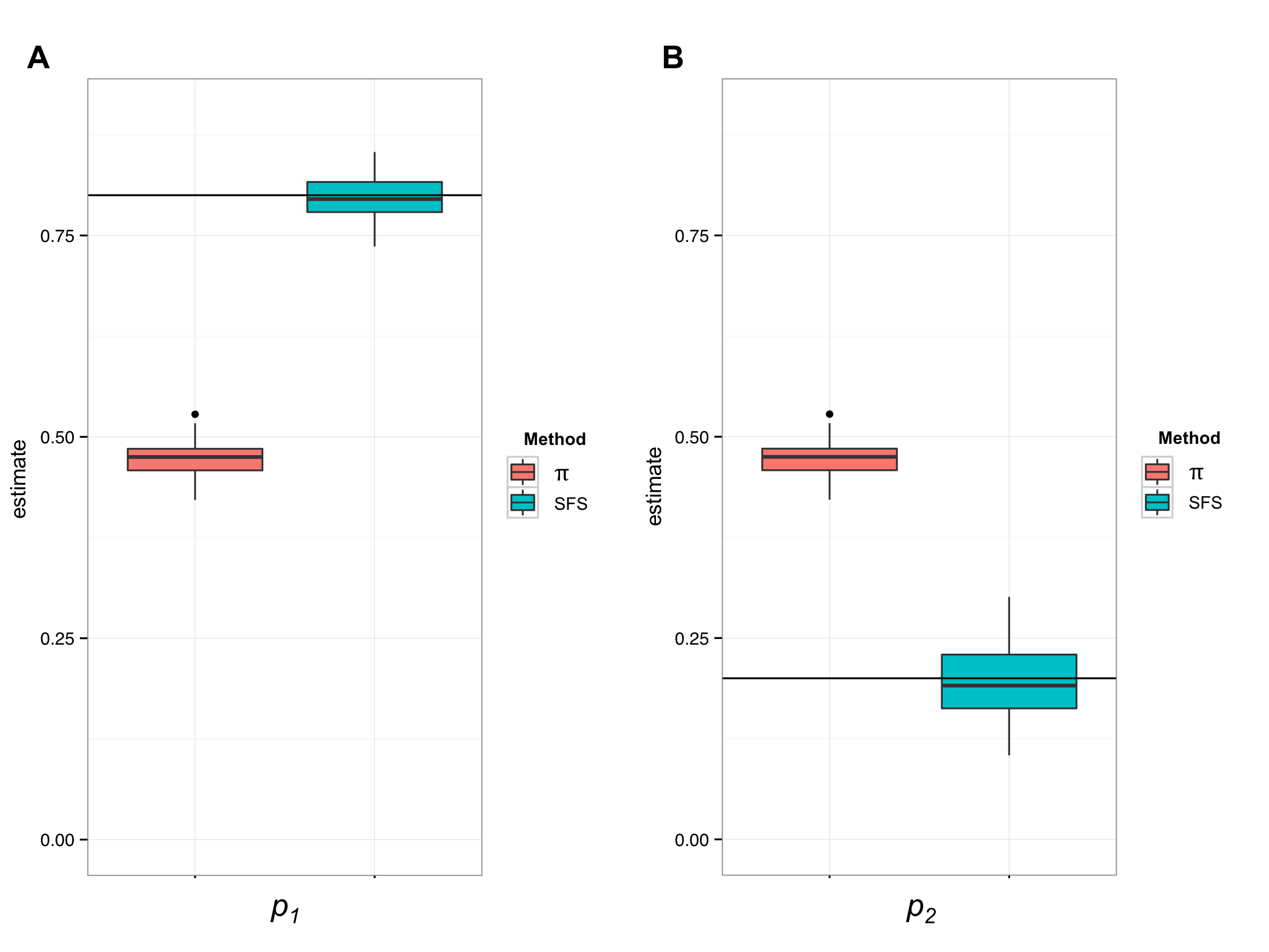

Supplement: S6 Fig — We show sex-bias estimators from simulations with a female bias outside the bottleneck (p1 = 0.8) and a male bias during the bottleneck (p2 = 0.2). Our estimator p˜ in green (“SFS”), recovers the true value of (A) p1 and (B) p2, denoted by the gray horizontal line. The estimator pπ in red (“π”), gives a single biased estimate. (TIF) [file pgen.1008293.s007.tif]

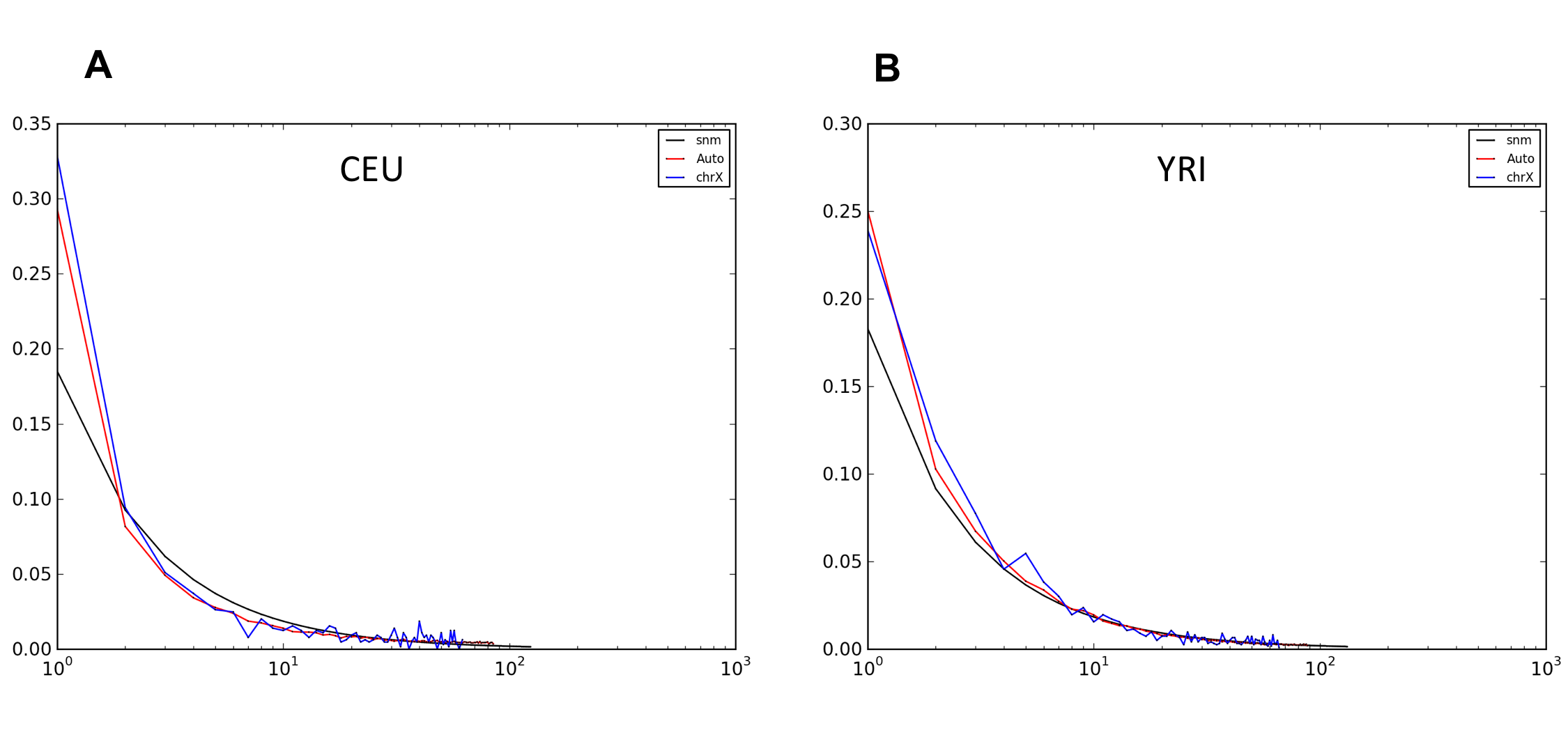

Supplement: S7 Fig — Folded site frequency spectra (SFS) for 1000 Genomes Project exomes of (A) Europeans (CEU) and (B) Yorubans (YRI). Autosomal SFS (red, “Auto”) were projected down to have the same dimensions as X-chromosomal SFS (blue, “chrX”). The expected SFS based on the standard neutral model is in dark gray (“snm”). (TIF) [file pgen.1008293.s008.tif]

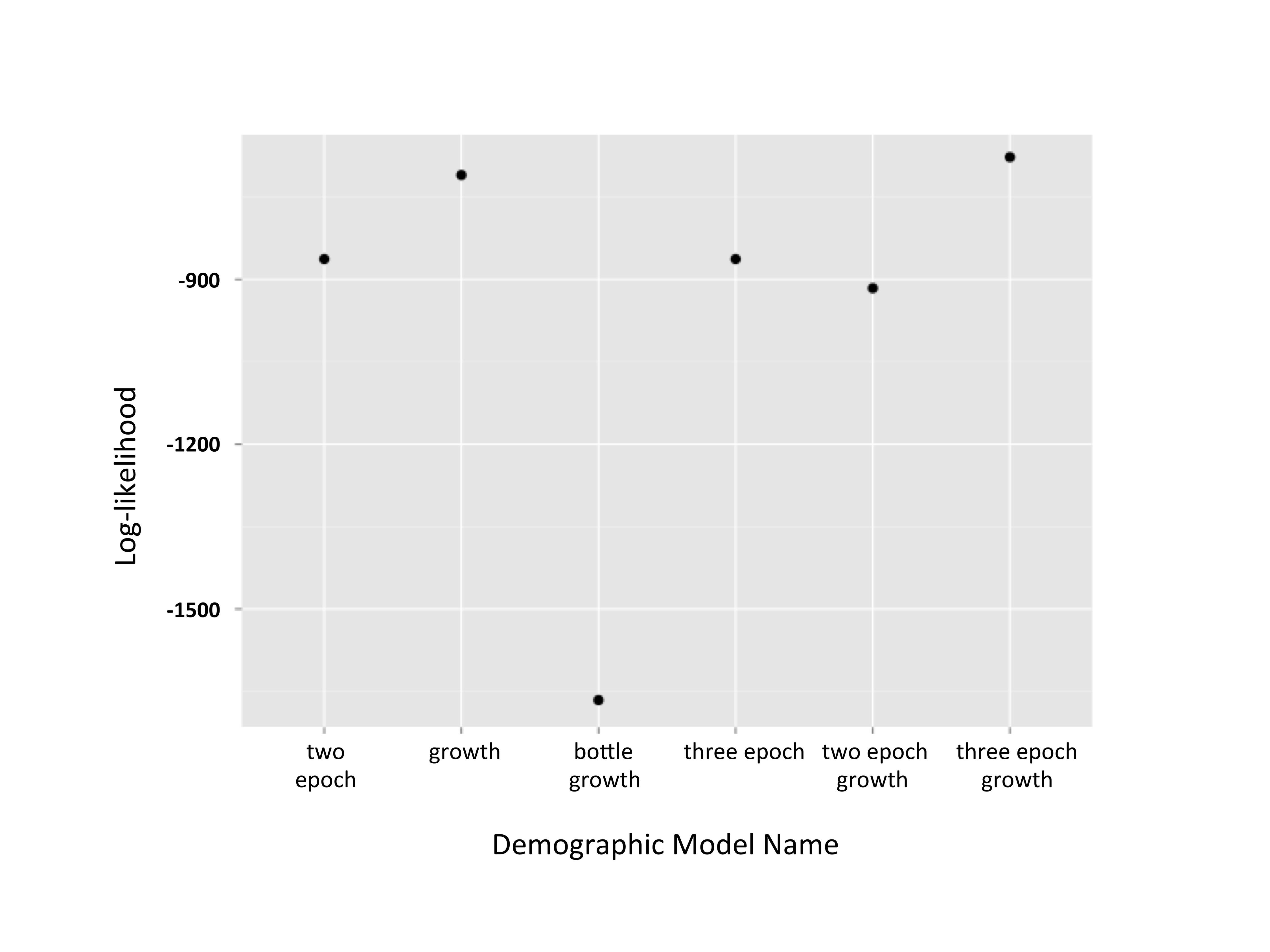

Supplement: S8 Fig — Autosomal demographic model log-likelihoods from 1000 Genomes Project European (CEU) whole-genome sequence data. The best-fitting model, a complex model (“three epoch growth”), has the largest log-likelihood. (TIF) [file pgen.1008293.s009.tif]

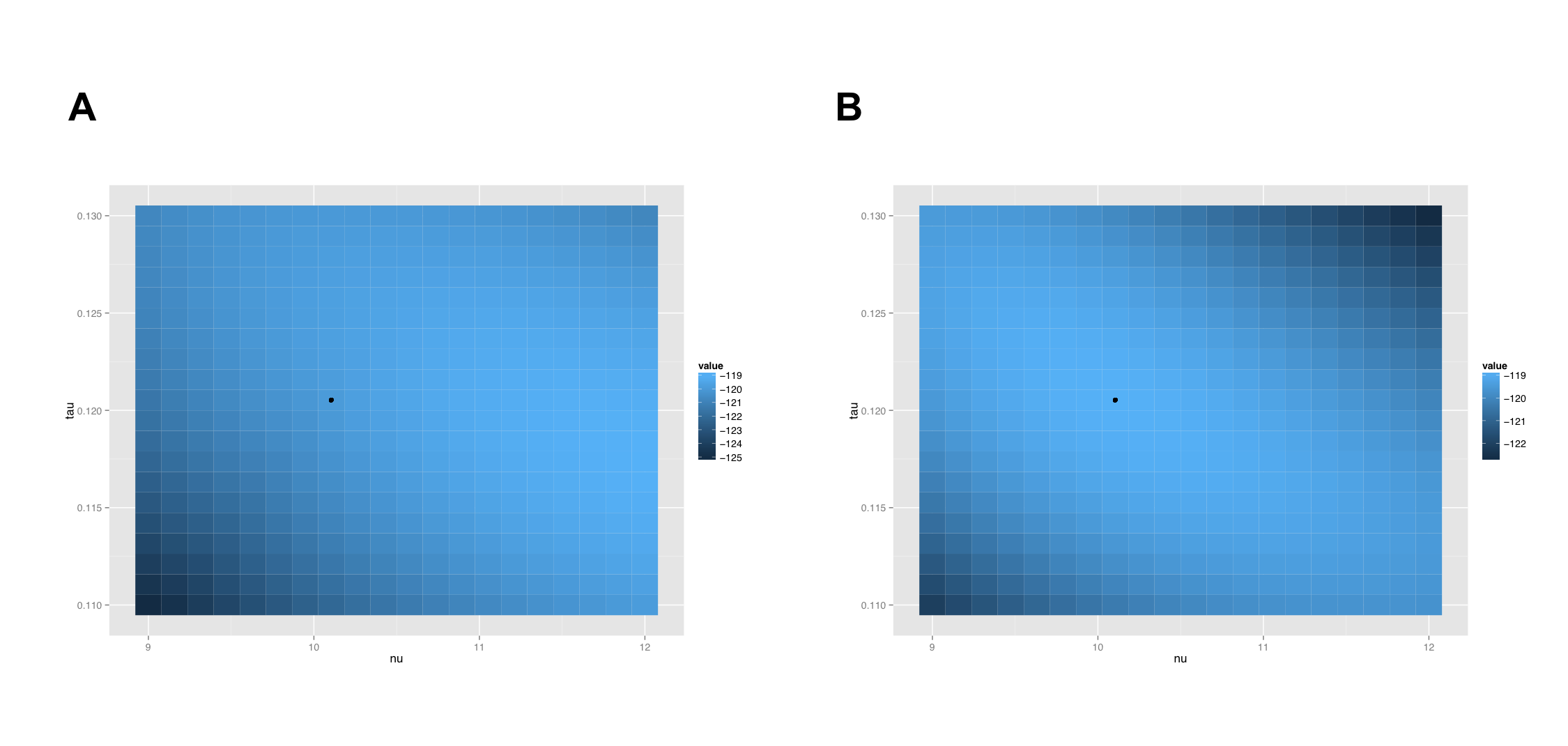

Supplement: S9 Fig — Log-likelihoods of data simulated under a two-epoch model for a grid of fold-size changes, ν (“nu”), on the x-axis and times, τ (“tau”), on the y-axis. The true simulation parameter is denoted by a black dot, light blue regions have better log-likelihoods, and dark blue regions have poorer log-likelihoods. (A) With the default ∂a∂i timescale parameter of 1e−3 and a coarse grid, the true point does not have the best likelihood. (B) With a smaller timescale parameter of 1e−4 and a finer grid, the true point has the best likelihood. (TIF) [file pgen.1008293.s010.tif]

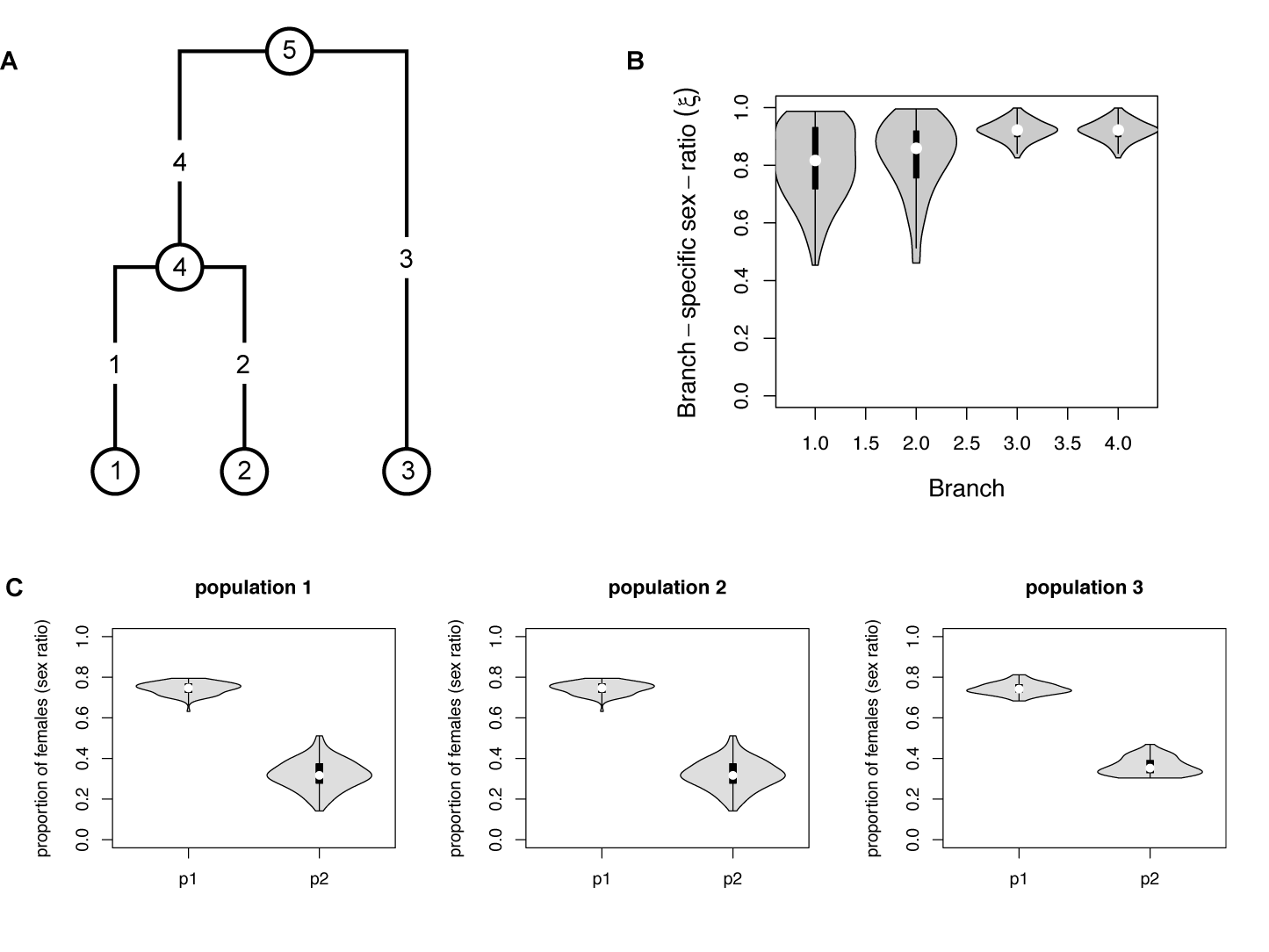

Supplement: S10 Fig — Data simulated from three populations, each of which experience a sex-biased bottleneck. (A) Multi-population tree model where populations are nodes. The contemporary populations 1, 2, and 3 are sampled. A male-biased bottleneck occurs on branches 3 and 4 where the proportion of females outside the bottleneck, p1, is 0.8 and the proportion of females during the bottleneck, p2, is 0.2. The proportion of females is 0.8 on branches 1 and 2. (B) KimTree estimates a female bias on each branch (ξ) and does not detect the male-biased bottlenecks because it cannot fit sex-bias parameters that change on a branch. (C) Our method correctly estimates a female bias outside the bottleneck (“p1”) and a male during the bottleneck (“p2”) in all three populations. (TIF) [file pgen.1008293.s011.tif]
